# Hematopoietic stem and progenitor cell proliferation and differentiation requires the trithorax protein Ash2l

**DOI:** 10.1038/s41598-019-44720-3

**Published:** 2019-06-04

**Authors:** Juliane Lüscher-Firzlaff, Nicolas Chatain, Chao-Chung Kuo, Till Braunschweig, Agnieszka Bochyńska, Andrea Ullius, Bernd Denecke, Ivan G. Costa, Steffen Koschmieder, Bernhard Lüscher

**Affiliations:** 1 Institute of Biochemistry and Molecular Biology, RWTH Aachen University, Pauwelsstrasse 30, 52074 Aachen, Germany; 2 Department of Hematology, Oncology, Hemostaseology, and Stem Cell Transplantation, RWTH Aachen University, Pauwelsstrasse 30, 52074 Aachen, Germany; 3 Institute for Computational Genomics, RWTH Aachen University, Pauwelsstrasse 30, 52074 Aachen, Germany; 4 Institute of Pathology, RWTH Aachen University, Pauwelsstrasse 30, 52074 Aachen, Germany; 5 Interdisciplinary Center for Clinical Research Aachen, Faculty of Medicine, RWTH Aachen University, Pauwelsstrasse 30, 52074 Aachen, Germany; 6 Present Address: QIAGEN GmbH, Qiagen Str. 1, 40724 Hilden, Germany

**Keywords:** Cell biology, Molecular biology

## Abstract

Post-translational modifications of core histones participate in controlling the expression of genes. Methylation of lysine 4 of histone H3 (H3K4), together with acetylation of H3K27, is closely associated with open chromatin and gene transcription. H3K4 methylation is catalyzed by KMT2 lysine methyltransferases that include the mixed-lineage leukemia 1–4 (MLL1-4) and SET1A and B enzymes. For efficient catalysis, all six require a core complex of four proteins, WDR5, RBBP5, ASH2L, and DPY30. We report that targeted disruption of *Ash2l* in the murine hematopoietic system results in the death of the mice due to a rapid loss of mature hematopoietic cells. However, lin^−^Sca1^+^Kit^+^ (LSK) cells, which are highly enriched in hematopoietic stem and multi-potent progenitor cells, accumulated in the bone marrow. The loss of Ash2l resulted in global reduction of H3K4 methylation and deregulated gene expression, including down-regulation of many mitosis-associated genes. As a consequence, LSK cells accumulated in the G2-phase of the cell cycle and were unable to proliferate and differentiate. In conclusion, Ash2l is essential for balanced gene expression and for hematopoietic stem and multi-potent progenitor cell physiology.

## Introduction

Cell identity is defined by differential gene expression^1,2^. This is achieved by the coordinated action of transcription factors and cofactors that control chromatin accessibility and the recruitment and activity of RNA polymerase complexes^3,4^. The smallest units of chromatin are nucleosomes, composed of core histones and DNA. Different enzymes can modify histones, thereby controlling protein-protein interactions, modulating access to DNA and regulating RNA polymerase loading and processivity^5,6^. A recent census suggests that more than 450 post-translational histone modifications exist^7^, one of them being methylation of lysine 4 of histone H3 (H3K4me). This modification is linked to open chromatin and in many situations with gene transcription. In particular, trimethylation (H3K4me3) and monomethylation (H3K4me1) are associated with promoters and enhancers, respectively, and thus have been suggested to possess important functions in controlling gene expression^8–10^.

Major contributors to methylation of H3K4 comprise the lysine-specific methyltransferases of the KMT2 family, consisting of MLL1 to 4 and SET1A and B (KMT2A-D, F and G, respectively). SET1A and B complexes preferentially trimethylate H3K4 at core promoters, whereas MLL3 and 4 predominantly mono- and dimethylate H3K4 in enhancer regions. MLL1 and 2 appear to be able to mono-, di- or trimethylate H3K4, depending on their recruitment to core promoters or enhancers^10,11^. Knockout (KO) studies of different KMT2 family members have demonstrated their importance for mouse development and tissue homeostasis^12–15^. Homozygous deletion of *Mll1* is embryonically lethal, whereas the *Mll1*^+/−^ heterozygotes show retarded growth and a number of additional abnormalities^12,16^. Mll1/KMT2A appears particularly relevant in hematopoietic stem and progenitor cells^17–20^, whereas Mll2/KMT2B is essential during early embryonic development^13^, and is required in oocytes and during spermatogenesis^21,22^. The molecular consequences are also distinct as e.g. different *Hox* genes are deregulated in *Mll1* and *Mll2* KO cells. Loss of Mll3/KMT2C and Mll4/KMT2D results in death around birth and day E9.5, respectively^14^. Set1A and B (KMT2F and G, respectively) are also essential, the former during gastrulation, while the *Set1B* KO embryos survive until day E11.5^15^. These findings suggest that each of the 6 KMT2 complexes is required for defined aspects of development and thus are at least in part functionally distinct.

For catalytic activity and for recruitment to chromatin KMT2 enzymes require the interaction with the WRAD complex, composed of WDR5, RBBP5, ASH2L, and two copies of DPY30^10,11,23^. Additional subunits are associated with distinct KMT2 complexes (aka COMPASS), further increasing diversity of these multi-protein cofactors^10,24^. WRAD components are essential as far as studied. Ash2l is required for early mouse development^25^ and for liver homeostasis^26^. Moreover, Dpy30 is essential during embryogenesis and critical for hematopoietic stem and progenitor cell differentiation^27–29^. In these studies, the heterozygous animals revealed no phenotype, suggesting that neither Ash2l nor Dpy30 is haploinsufficient. In summary, KMT2 complexes exert critical functions in mouse development and in organ homeostasis^11,23,30^.

Epigenetic modifications of DNA and core histones play prominent roles in the development of hematopoietic malignancies, such as myeloid leukemia and aggressive lymphomas, and the corresponding writers, readers and erasers are considered as drug targets^30–32^. The association of KMT2 complexes with cancer has been well documented and is particularly evident for *MLL1* as translocations of this gene are associated with acute leukemias^33^. Other KMT2 methyltransferases have been linked to other malignancies (see e.g.^34–37^). An involvement of ASH2L in tumorigenesis has also been suggested. We have identified ASH2L as an 86 kDa interaction partner of the oncoprotein c-MYC^38^. Subsequently, ASH2L was found to cooperate with Ha-RAS in the transformation of rat embryo fibroblasts^39^. MYC is deregulated in the majority of hematopoietic malignancies^40^, and, together with ASH2L and other cofactors such as CBP/p300, regulates chromatin and gene transcription^41–43^. Furthermore, ASH2L interacts with MLK1 (megakaryocytic leukemia-1), a transcription factor originally identified in acute megakaryocytic leukemia and subsequently shown to affect megakaryocytic, monocytic, and granulocytic differentiation and function^44–46^. Moreover, low expression of ASH2L has been correlated with increased survival of patients with acute myeloid leukemia^47^. Beyond hematopoiesis, ASH2L is overexpressed in the majority of human tumors and its knockdown interferes with H3K4 methylation and tumor cell proliferation^39,48–50^. Together, these data suggest an important role of ASH2L for the differentiation and proliferation of hematopoietic cells both under physiologic conditions as well as during malignant transformation.

To understand the function of Ash2l in the hematopoietic system in more detail, we generated conditional *Ash2l* KO mice using the Mx1-Cre/loxP system. The loss of Ash2l protein expression in the hematopoietic system led to a differentiation block of early hematopoietic progenitor cells. This block was associated with a late cell cycle arrest. Consistent with this phenotype, genes encoding factors associated with G2/M-phase progression were down-regulated upon loss of Ash2l. The consequence of this differentiation block is severe pancytopenia with subsequent death of the animals.

## Results

### Mx1-Cre-dependent knockout of *Ash2l* is lethal and prevents differentiation of hematopoietic cells

**Figure 1:**
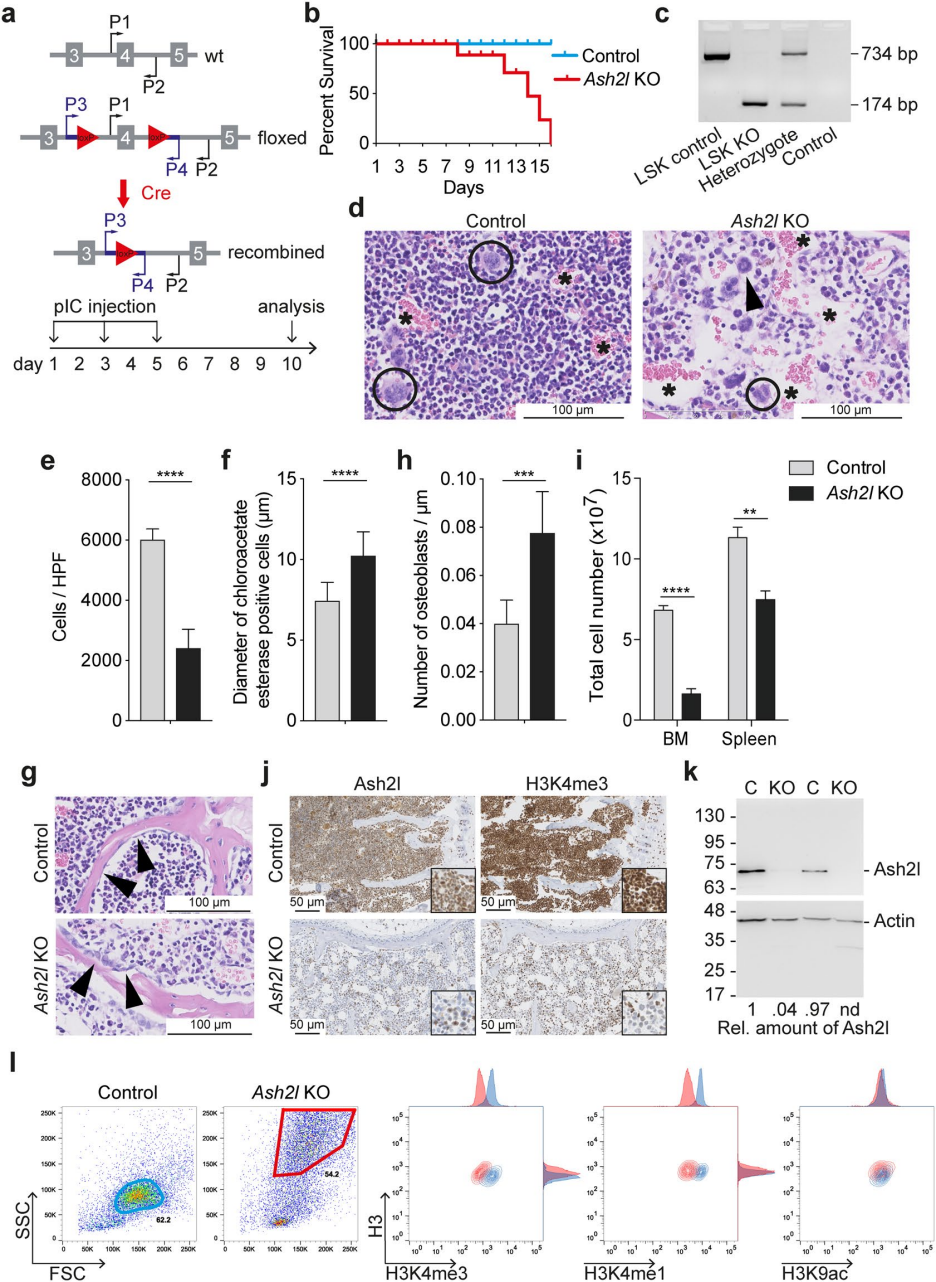
Ash2l is essential and its loss interferes with histone H3 lysine 4 methylation. (**a**) Scheme of the wild type (wt) and floxed exon 4, and the recombined *Ash2l* locus. The primers used to analyze the different alleles are indicated. Drawing not to scale. The experimental design is shown at the bottom. (**b**) Effect of pIC treatment on *Ash2l* KO (n = 8) and control (n = 2) mice. Kaplan-Meier plot from the experimental endpoint. (**c**) PCR analysis of LSK DNA (mice treated for 10 d) using primers P3/P4 (panel a). LSK control, pool of six wt animals; LSK KO, pool of six *Ash2l*^fl/fl^: *Mx1-Cre* KO animals; heterozygote, tail DNA of an *Ash2l*^wt/fl^: *CAG-CreER*^+^ animal; control, no template. Floxed and recombined *Ash2l* alleles with 734 and 174 bp, respectively. (**d**) Sections of fixed sterna from control and *Ash2l* KO animals stained with H&E. Circles, megakaryocytes; Arrow head, granulocyte; *sinuses. (**e**) Total number of cells determined from 24 control and 40 KO HPF sternal sections of 4 animals each. (**f**) Diameters were measured of 100 chloroacetate esterase positive cells in sternal sections of 2 control and 2 KO animals. (**g**) H&E stainings of sternal sections containing areas of trabecular bone are displayed. Arrow heads indicate osteoblasts. (**h**) The length of the lining of enclosed fields of BM was measured and the number of osteoblasts determined (six areas from two animals each). (**i**) Total number of cells recovered from BM (2 tibias and 2 femurs/mouse) and from spleen (n = 3 each). (**j**) Serial tissue sections from fixed sterna stained with anti-Ash2l or anti-H3K4me3 antibodies as indicated. (**k**) Western blot analysis of proteins of BM cell lysates (from control (C) and *Ash2l* KO mice). Upper and lower halfs of the blot was probed for Ash2l and actin, respectively. (**l**) Flow cytometry analysis of fixed BM cells double-stained for histone H3 and H3K4me3, H3K4me1 or H3K9ac as indicated. Forward and side scatter plots (FSC and SSC, respectively) define the main population of control (blue gate) and *Ash2l* KO cells (red gate). These were assessed for histone H3 and the indicated histone marks. (****p < 0.0001; ***p < 0.001; **p < 0.01).

We generated mice with alleles of *Ash2l* harboring a floxed exon 4 and an Mx1-Cre transgene whose expression was stimulated by the intraperitoneal injection of the synthetic RNA analog polyinosinic-polycytidylic acid (pIC) (Fig. 1a)^51^. *Ash2l*^fl/fl^: *Mx1-Cre* animals were affected starting at day 8 upon pIC treatment and had to be sacrificed subsequently (Fig. 1b). In the following experiments, we analyzed animals and cells at day 10. Activation of Cre led to efficient recombination of the floxed sequences (Fig. 1c). Histological examination of the bone marrow (BM) in the sternum by hematoxylin&eosin (H&E) staining revealed a reduced cellularity in the *Ash2l* KO mice (Fig. 1d). The BM was populated less than half in *Ash2l* KO vs. control mice (Fig. 1e). We observed that all lineages of blood-forming cells were affected with the appearance of dysmorphic megakaryocytes, showing lobulated nuclei and reduced amounts of cytoplasm (Fig. 1d, circles). In granulopoesis, a higher number of ring-like myelocytes (band granulocytes) and metamyelocytes was visible (Fig. 1d, arrow head). This is consistent with the larger size of chloroacetate esterase stained cells in the *Ash2l* KO compared to control animals (Fig. 1f). We did not observe any obvious morphological differences for erythropoesis (Fig. 1d). In addition, the sternal sections of KO animals revealed widened sinuses (Fig. 1d, asterisks) and an altered number and morphology of osteoblasts. In control animals, these were spindle shaped, while in the *Ash2l* KO mice osteoblasts were heterogenous and often showed an enlarged and activated morphology (Fig. 1g, arrow heads). These cells stained positive for Ash2l. The number of osteoblasts lining the trabecular bones was increased significantly in the *Ash2l* KO vs control animals (Fig. 1h). The reduced cellularity in the BM was consistent with a roughly 4-fold decrease in total BM cells flushed from tibia and femur from *Ash2l* KO mice compared to controls (Fig. 1i; BM refers to the cells flushed from 2 tibias and 2 femurs/mouse). We also observed a decrease of hematopoietic cells isolated from spleen when KO animals were compared to control mice (Fig. 1i).

While essentially all cells in the BM of control animals were positive for Ash2l and for H3K4me3, roughly half of the cells were negative for these markers in *Ash2l* KO mice (Fig. 1j). This was particularly true for hematopoietic cells, whereas mesenchymal cells were Ash2l positive (overall 43.7 ± 6.1% Ash2l positive cells in *Ash2l* KO sternal sections). The decrease of Ash2l staining was consistent with a strong decrease of Ash2l protein of lysates of total BM-derived cells (Fig. 1k). Moreover, using intracellular flow cytometry, H3K4me3 and H3K4me1 were reduced in BM-derived cells, while H3K9ac was unchanged (Fig. 1l). Thus, the loss of Ash2l interfered with H3K4 methylation and correlated with reduced cell numbers in the BM.

**Figure 2:**
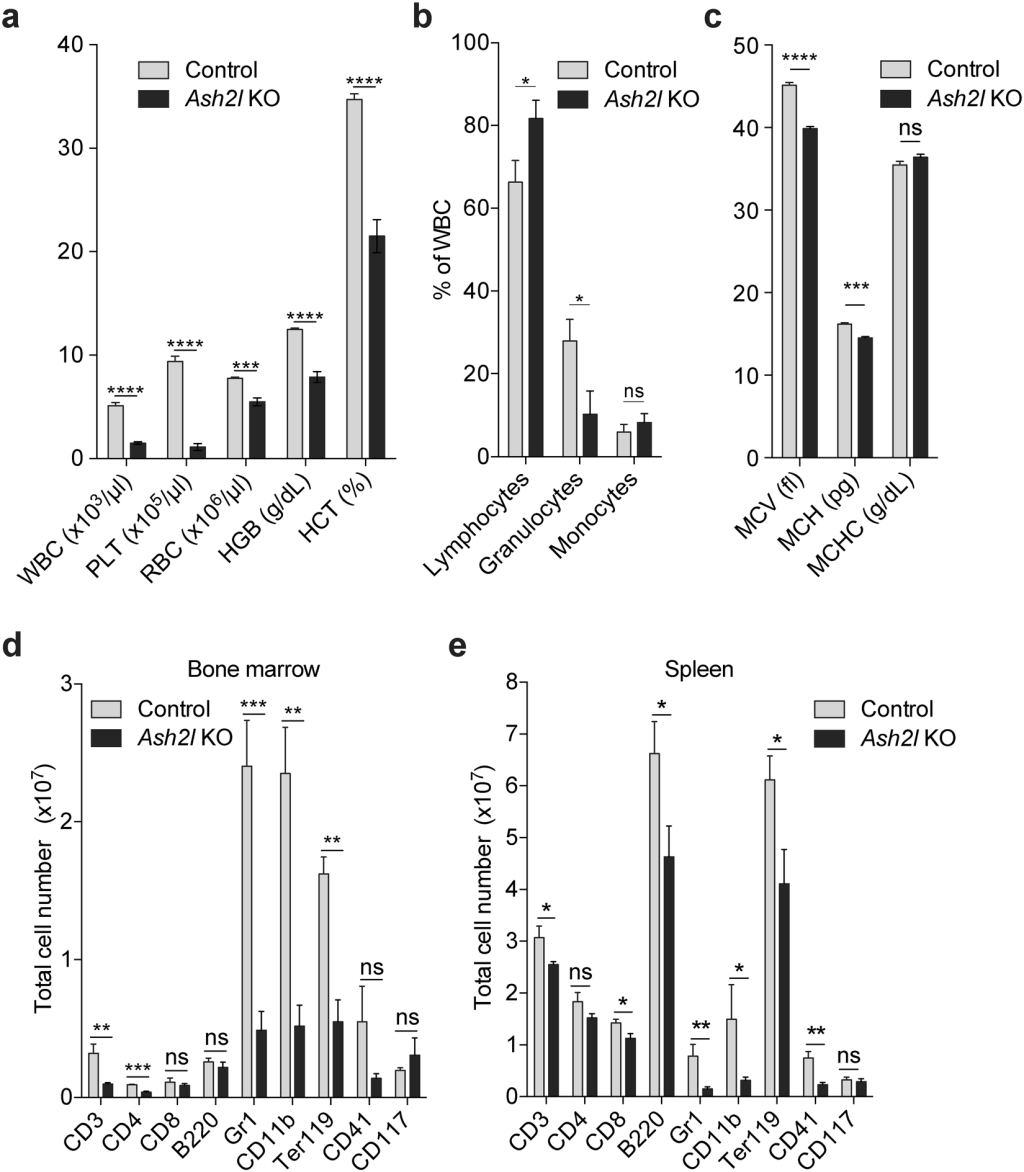
The conditional knockout of *Ash2l* interferes with adult hematopoiesis. (**a**) Whole blood from control (n = 49) and *Ash2l*^fl/fl^: *Mx1-Cre* KO (n = 35) mice was analyzed. WBC, white blood cells; PLT, platelets; RBC, erythrocytes; HGB, hemoglobin; HCT, hematocrit. (**b**) Blood count of lymphocytes, granulocytes, and monocytes from control (n = 7) and *Ash2l* KO (n = 6) mice. (**c**) Erythrocyte parameters from control and KO mice as in (panel a). MCV, mean corpuscular volume; MCH, mean corpuscular hemoglobin; MCHC, mean corpuscular hemoglobin concentration. (**d**) Flow cytometry analysis of BM cells from control (n = 3) and KO (n = 5) mice. Cells were labeled with lineage specific antibodies as indicated: CD3, T cells; CD4, helper T cells; CD8, cytotoxic T cells; B220, B cells; Gr1, granulocytes; CD11b, granulocytes/monocytes; Ter119, erythrocytes; CD41, megakaryocytes/platelets and myelo-erythoid progenitors; CD117, c-Kit positive cells. (**e**) Flow cytometry analysis of spleen cells from control (n = 3) and pIC treated KO (n = 5) mice as in (panel d). (****p < 0.0001; ***p < 0.001; **p < 0.01; *p < 0.05; ns, not significant).

The findings described above suggested severe defects in the hematopoietic system in response to the loss of Ash2l. We also noticed that the limbs of the pIC treated *Ash2l*^fl/fl^: *Mx1-Cre* animals were pale, indicative of a reduced number of erythrocytes and/or hemoglobin in the blood. Indeed, erythrocytes (RBCs), consistent with a reduced hematocrit and a decreased concentration of hemoglobin, as well as white blood cells (WBC) and platelets were decreased in peripheral blood (Fig. 2a). When the WBC were further itemized, we measured a percentage increase in lymphocytes and decrease in granulocytes, while little change for monocytes (Fig. 2b). This shift might reflect the lifetime differences of the various subpopulations of cells in the peripheral blood. The mean corpuscular volume of erythrocytes was reduced, while the corpuscular amount and concentration of hemoglobin were slightly affected (Fig. 2c). The rather small effect on RBCs is consistent with their half-life of between 22 and 42 days^52–55^. In addition to the peripheral blood, we isolated the cells from BM and spleen and quantified the different populations using surface markers. Most subpopulations in the BM were reduced (Fig. 2d). This was particularly obvious for short-lived granulocytes (Gr1^+^) and granulocytes/monocytes (CD11b^+^), while more long-lived T and B cells (e.g. CD8 and B220, respectively) were less affected. The same tendency was observed in the spleen (Fig. 2e). Thus, Ash2l loss blocked hematopoietic cell proliferation and/or differentiation resulting in the death of the animals.

### Lin^−^Sca-1^+^Kit^+^ stem and precursor cells increase upon Ash2l loss

**Figure 3:**
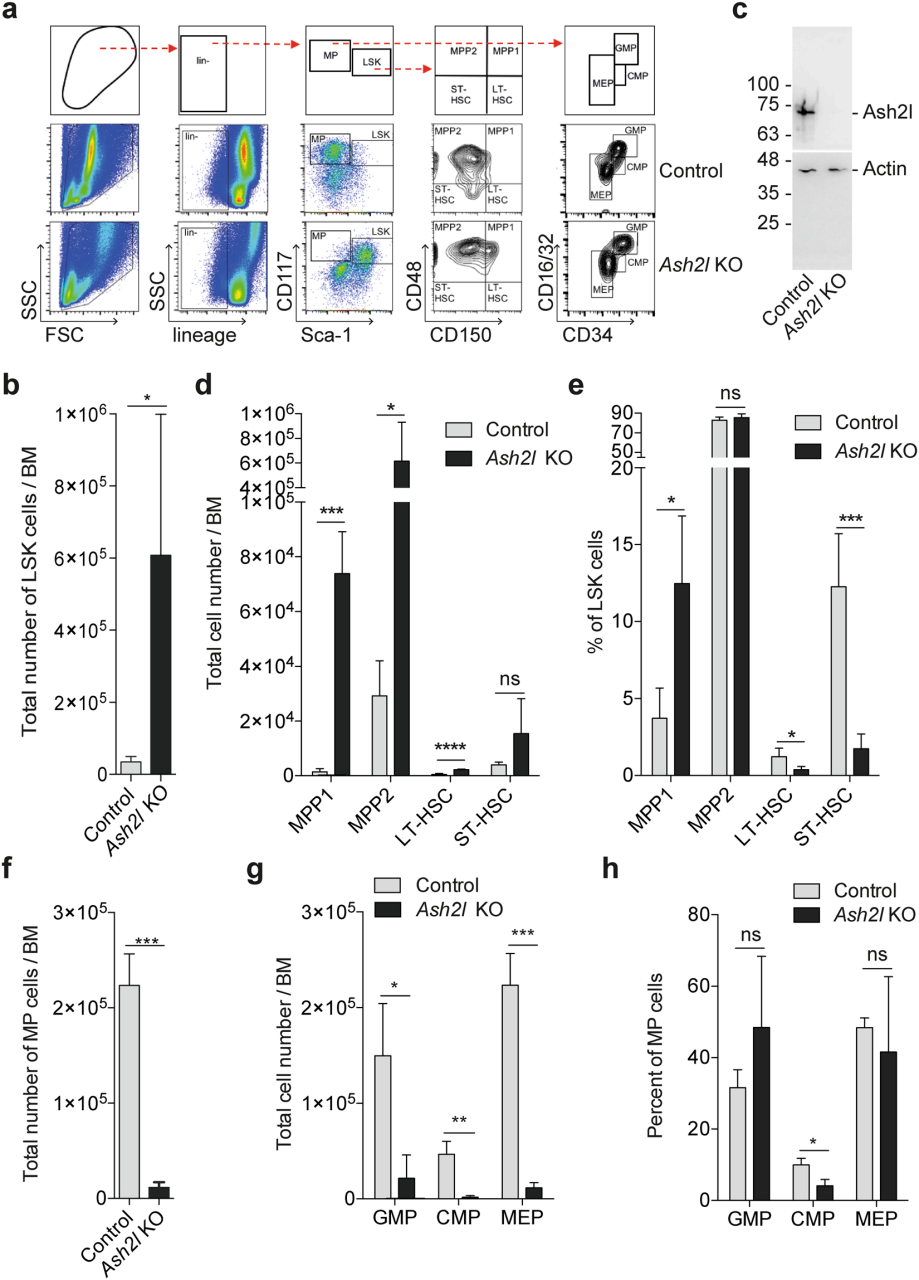
Accumulation of LSK cells upon the loss of Ash2l. (**a**) Scheme of the gating strategy (top row) and one representative example of BM cells from a control and an *Ash2l* KO mouse. (**b**) Number of LSK cells per BM from control (n = 3) and KO (n = 6) mice. (**c**) Western blot of 1.5 × 10^5^ LSK cells from a control and a KO mouse. (**d**,**e**) Number and percentage of different LSK cell sub-populations per BM from control (n = 3) and KO (n = 5) mice. (**f**) Number of myeloid progenitors (MP) per BM from control and KO mice (n = 3 each). (**g**,**h**) Number and percentage of different MP cell sub-populations per BM (n = 3 each). GMP, granulocyte macrophage progenitors; CMP, common myeloid progenitors; MEP, megakaryocyte erythroid progenitors. (****p < 0.0001; ***p < 0.001; **p < 0.01; *p < 0.05; ns, not significant).

*Ash2l* KO BM cells were larger and displayed a higher degree of granularity than control cells (Figs 1l and 3a). A more detailed analysis revealed a strong increase in the total number of lineage negative LSK cells (lin^−^Sca-1^+^Kit^+^), which contain hematopoietic stem and multi-potent progenitor cells (HSCs and MPPs, respectively) (Fig. 3a,b). Because we expected broad effects on gene expression in response to a loss of Ash2l, genes of relevant markers used for the classification of BM cells (as shown in Fig. 3) were analyzed (see Table S1, deposited data GSE114433). Of note, the expression of *Sca-1* in LSK cells was increased 2.5-fold, whereas the genes of other markers, including CD16, CD32, CD34, CD117, and CD150, varied below 1.5-fold. Thus, the effects on the distribution of LSK and MP cells are most likely very limited. In LSK cells purified by fluorescence-activated cell sorting (FACS), an almost complete loss of Ash2l was observed (Fig. 3c), supporting the BM analysis (Fig. 1). Three LSK subpopulations increased, i.e. long-term (LT; lin^−^Sca-1^+^cKit^+^CD48^−^CD150^+^) repopulating HSCs as well as MPP1 (lin^−^Sca-1^+^cKit^+^CD48^+^CD150^+^) and MPP2 (lin^−^Sca-1^+^cKit^+^CD48^+^CD150^−^) cells, while the short-term (ST; lin^−^Sca-1^+^cKit^+^CD48^−^CD150^−^) repopulating HSCs showed a trend towards increased cell numbers (Fig. 3a,d)^56,57^. This was most prominent for MPPs when comparing absolute numbers (Fig. 3d). However, the relative contribution of LT- and ST-HSCs to the LSK population was reduced in *Ash2l* KO animals (Fig. 3e). This increase in LSK cells, and in particular the more differentiated MPPs, suggested that the hematopoietic system responded to the reduction of mature cells by promoting early proliferation and differentiation of stem and progenitor cells^58,59^. However, the more differentiated myeloid progenitors (MP; lin^−^Sca-1^−^cKit^+^) were almost completely lost in the BM (Fig. 3a,f). The total cell number of common myeloid, granulocyte/macrophage, and megakaryocyte erythroid progenitors (CMPs, GMPs, and MEPs, respectively) was strongly reduced in *Ash2L* KO mice, while the relative proportion remained similar (Fig. 3g,h). The exception was CMPs, which were significantly reduced, likely reflecting that these cells differentiated into GMPs and MEPs, but further development was stopped. These findings suggest that LSKs, in particular MPPs, accumulate but cannot replenish the MP subpopulations in the absence of Ash2l.

### Genes associated with mitosis are downregulated in *Ash2l* KO LSK cells

**Figure 4:**
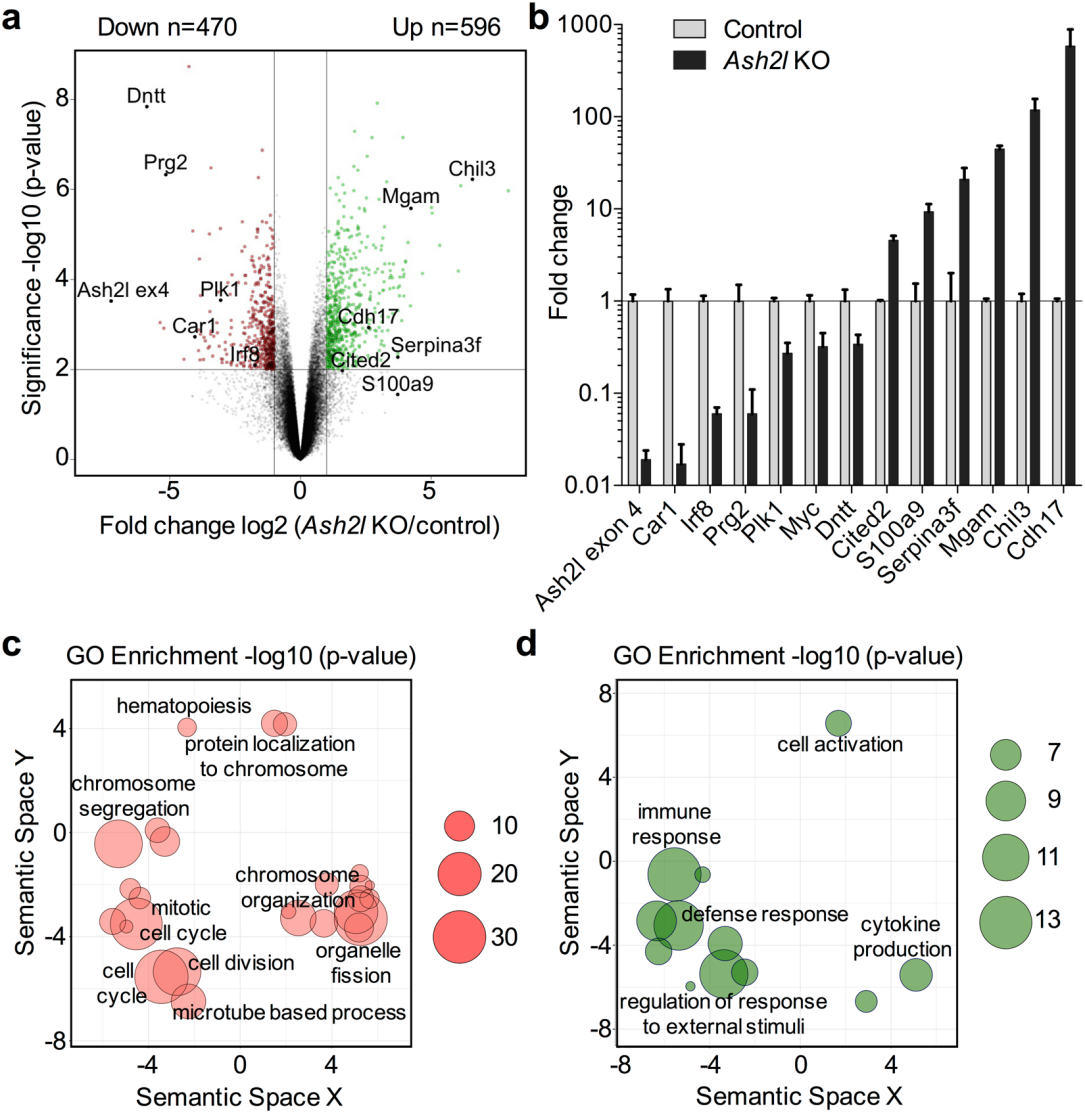
The loss of Ash2l protein expression deregulates gene expression. (**a**) Total RNA of LSK cells (n = 3 each) was analyzed on microarrays. A volcano plot was employed to visualize differences in gene expression. (**b**) RT-qPCR analysis of selected genes from (**a**) using RNA prepared from LSK cells of biological replicates. (**c**,**d**) GO analysis of down- and up-regulated genes (panels c and d, respectively) with the indicated p-values (circle size). The semantic space represents the similarities of GO terms, i.e. GO terms annotated with similar genes have similar values. Only representative GO terms are shown.

The *in vivo* analysis of hematopoietic cells in response to the loss of Ash2l suggested strong effects on genes associated with proliferation and differentiation. Deregulated gene expression is expected considering the known function of Ash2l in KMT2 methyltransferase complexes and with the observed reduction of H3K4 methylation in *Ash2l* KO BM and LSK cells (Figs 1 and 3). Therefore, we analyzed RNA isolated from FACS-sorted LSK cells of control and *Ash2l* KO animals on microarrays. This revealed 470 down- and 596 up-regulated genes by >2-fold and a p-value < 0.01 in three independent experiments (Fig. 4a). For control the *Ash2l* RNA with deleted exon 4 was measured and found to be down-regulated more than 100-fold in the knockout compared to control cells (Fig. 4a). The changes in expression of the deregulated genes was considerable and verified by RT-qPCR for selected genes (Fig. 4b). Again, the loss of exon 4 containing *Ash2l* RNA was more than 50-fold, supporting the array data and indicating that the knockout was efficient (Fig. 4a,b). Of note is that the verified genes showed C_T_ values of smaller than 30, except for Cdh17 with a C_T_ value of 32 in control cells, in the RT-qPCR analysis (Fig. 4b). Thus, the expression of all verified genes was easily measured. This is particularly relevant for the up-regulated genes, indicating that their enhanced expression was not an artifact of very low expression in the controls. Gene ontology (GO) analysis (p-value < 0.05) of down-regulated genes revealed a very strong association with processes late in the cell cycle, i.e. in particular with G2/M-phase progression (Fig. 4c and Table S4)^60^. For example, the genes encoding the key G2/M-phase transition kinases PLK1 (polo-like kinase 1), Aurora A kinase, and CDK1 (Cyclin-dependent kinase 1) were down-regulated (see Table S1 for online access to the array data). The GO terms for up-regulated genes were less focused and included a broad range of terms (Fig. 4d and Table S5).

### Bone marrow cells of *Ash2l* KO animals are unable to proliferate

**Figure 5:**
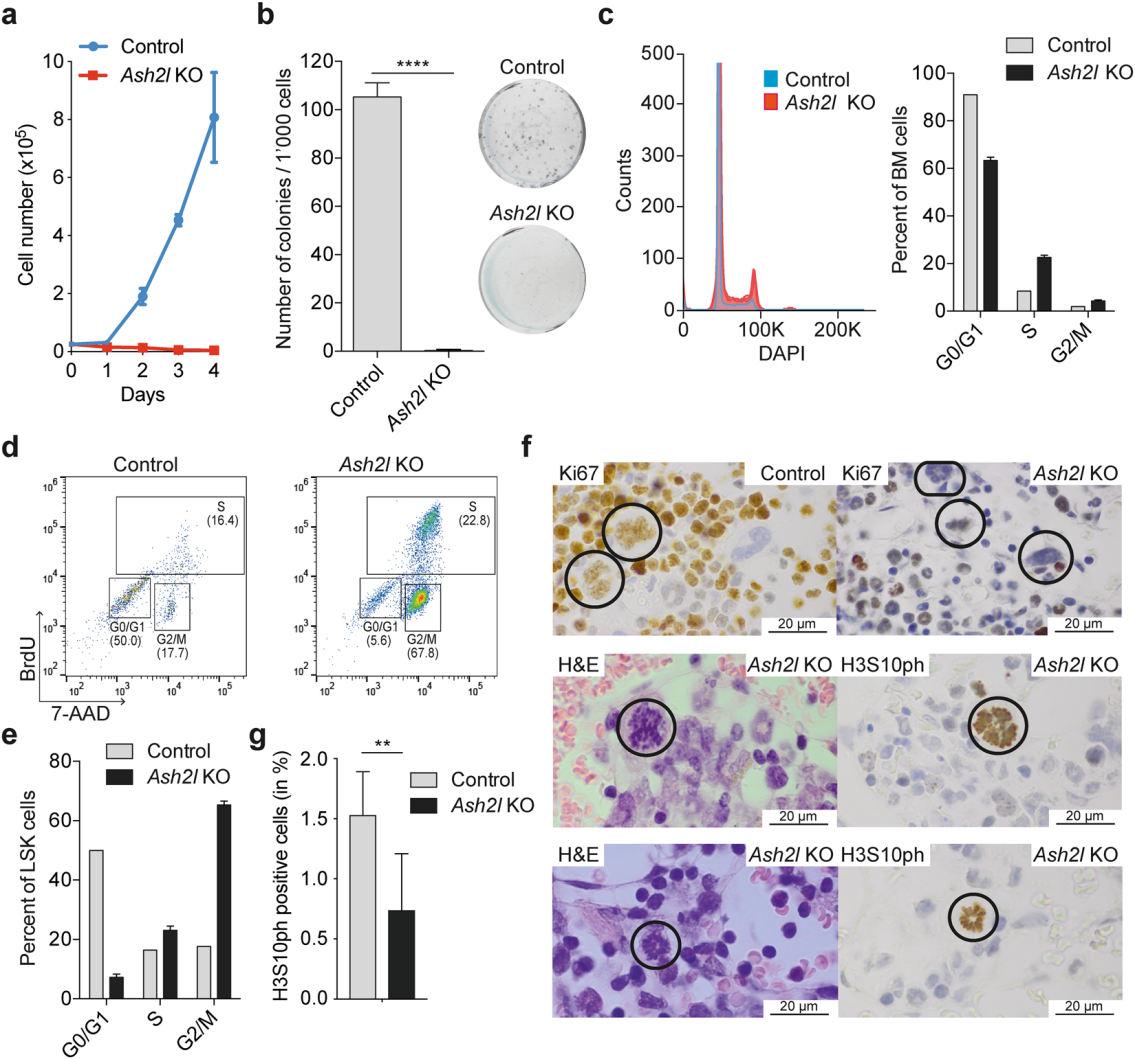
G2/M-phase arrest of *Ash2l*^−/−^ LSK cells. (**a**) Proliferation of LSK cells from control (pool of 5) and KO (n = 4) mice (each seeded in triplicates) as determined by cell counting. (**b**) Colony formation of LSK cells in semi-solid media. Representative plates of control and KO cells are shown (right). The number of colonies was counted (n = 4 each, seeded in triplicates). (**c**) Flow cytometry analysis of DAPI stained BM cells of control (pool of 3) and KO (n = 3) mice. A histogram and a summary of the cell cycle distribution are shown. (**d**) Flow cytometry analysis of LSK cells labeled *in vivo* with BrdU and stained *in vitro* with 7-AAD and anti-BrdU antibodies from a pool of 3 wt animals (control) and an *Ash2l* KO mouse. (**e**) Summary of the BrdU/7-AAD cell cycle flow cytometry analysis of control (pool of 3) and KO (n = 3) mice. (**f**) Sections of fixed sterna from control and *Ash2l* KO animals stained with H&E and immunohistochemically for Ki67 and histone H3 phosphorylated serine 10 (H3S10ph) as indicated. Top panels, indicated are megakaryocytes stained for Ki67 in the control, not stained in the KO samples. Middle panels, defective mitosis in a megakaryocyte. Bottom panels, defective mitosis in granulocytes. (**g**) Summary of mitotic cells stained for H3S10ph. 20–30 HPFs of 3 control and KO animals each were analzyed, corrected for the number of cells present per section. (****p < 0.0001; **p < 0.01).

The gene expression analysis suggested a proliferation defect in the LSK cells. Therefore, we assessed the properties of FACS-sorted *Ash2l* KO LSK cells. While the control cells expanded efficiently in liquid culture, the *Ash2l* KO cells did not proliferate (Fig. 5a). Similarly, these cells were unable to form colonies in semi-solid media (Fig. 5b), despite the accumulation of these LSK cells in the mouse. Because of the strong association with cell cycle regulatory genes, we analyzed the cells by flow cytometry. We observed an increase of *Ash2l* KO total BM cells in the S- and G2/M-phases of the cell cycle (Fig. 5c). Because the BM contains also more mature hematopoietic cells that are preferentially in the G0/G1-phase of the cell cycle, FACS-sorted LSK cells were analyzed. To detect LSK cells that are synthesizing DNA and thus are in the S-phase of the cell cycle, mice were injected with BrdU 2 h prior to harvest. In addition, the isolated cells were stained with 7-aminoactinomycin D (7-AAD) to measure DNA content. These flow cytometry analyses revealed an increase in S-phase and particularly in G2/M-phase cells (Fig. 5d). Indeed, the majority of the LSK cells of *Ash2l* KO animals accumulated in the G2/M-phase of the cell cycle, while very few cells were in G0/G1 (Fig. 5e). This indicated, together with the inability to proliferate and the down-regulation of G2/M-phase regulators, that most of the LSK cells accumulated late in the cell cycle. The lack of proliferation, despite being mainly in S and G2/M, was also suggested by a reduction in Ki67 staining from 80–90% in control to 40–50% in KO sections (Fig. 5f). To discriminate between G2 and M, we determined the number of mitotic cells by H&E and anti-histone H3 serine 10 phosphorylation (H3S10ph) staining. Mitotic cells were decreased 2-fold in BM cells of *Ash2l* KO vs control animals (Fig. 5g). We noted that many mitotic figures in the KO animals were abnormal with round, astral appearance of condensed chromosomes that is not observed in normal BM. This was evident in megakaryocytes and granulocytes (Fig. 5f, middle and bottom panels, respectively). Our previous study on the cooperativity of ASH2L with oncogenic Ha-RAS had also revealed altered mitotic figures^39^. The star-like arrangement of mitotic chromosomes is reminiscent of a monopolar spindle^61^, suggesting that Ash2l is important for the control of mitosis.

**Figure 6:**
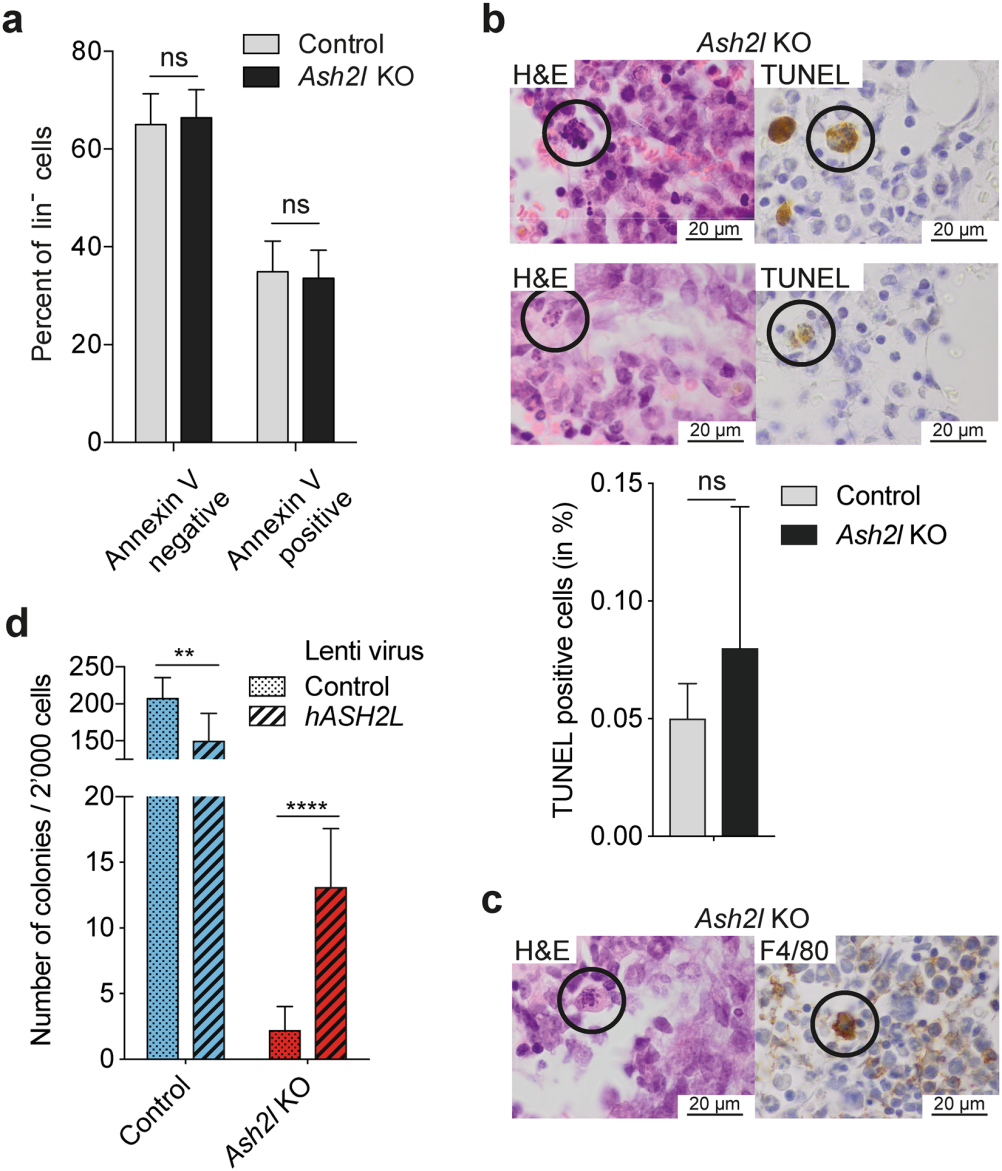
No increase in apoptosis in *Ash2l*^−/−^ cells. (**a**) Annexin V staining of lineage negative (lin^−^) cells isolated from BM of control and KO animals (n = 4 each). (**b**) Sections of fixed sterna from *Ash2l* KO animals stained with H&E and with TUNEL as indicated. The top panels show apoptotic megakaryocytes, the middle panels apoptotic granulocytes (circles). The bottom panel summarizes TUNEL positive cells from 10 HPFs of 2 control and 2 KO animals, corrected for the number of cells present in each section. (**c**) Sections of fixed sterna from *Ash2l* KO animals stained with H&E and immunohistochemically for F4/80 as indicated. Circles indicate macrophages. (**d**) Complementation with human ASH2L. Lin^−^ cells (n = 3 each) were infected with control or hASH2L-expressing virus, seeded in semi-solid media and the number of colonies was counted. (****p < 0.0001; **p < 0.01; ns, not significant).

Of note is, that we did not observe an increase in apoptotic cells upon loss of Ash2l, measured either by determining the sub-G0/G1 cells or by staining for Annexin V (Figs 5c and 6a). While the staining with DAPI revealed few sub-G0/G1 cells (Fig. 5c), roughly 30% of lin^−^ cells isolated from total BM were annexin V positive (Fig. 6a). To further evaluate apoptotic cells in the tissue, sternal sections were analyzed by TdT-mediated dUTP-biotin nick end labeling (TUNEL). We observed apoptotic megakaryocytes and granulocytes both with H&E and TUNEL staining (Fig. 6b, top and bottom panels, respectively). Quantification revealed small numbers of TUNEL positive cells in control and KO animals, with a tendency for more staining in the latter (Fig. 6b). The small number of apoptotic cells was in line with only few debris-loaded macrophages (1–2/10 HPFs) seen in the *Ash2l* KO sternum sections (Fig. 6c). Together, our findings suggest strongly that a major consequence of a loss of Ash2l in LSK cells is not apoptosis but an inability to proliferate because of a G2-phase cell cycle arrest.

Finally, we addressed whether the lineage negative (lin^−^) BM cells of *Ash2l* KO animals can be complemented by human ASH2L. The cells were infected with a lentivirus expressing ASH2L or with a control virus and seeded for colony formation assays. While ASH2L overexpression in control cells slightly reduced the number of colonies, a significant increase of colonies in *Ash2l* KO cells was observed (Fig. 6d). The numbers of rescued cells were small, probably reflecting that the cells could not be rescued anymore. Nevertheless, this experiment demonstrates that the key factor lacking was Ash2l and that human ASH2L was able to compensate for the murine protein. Thus, Ash2l is necessary for the proliferation of BM-derived hematopoietic cells.

## Discussion

The trithorax protein Ash2l is a core component of KMT2 complexes that methylate histone H3K4^10,11^. Previously, it has been demonstrated that *Ash2l* KO embryos do not develop beyond the implantation state^25^. Moreover, liver disintegration was observed in response to loss of Ash2l^26^. Our findings using an Mx1-Cre inducible *Ash2l* KO model revealed that Ash2l is also necessary for maintaining adult hematopoiesis. Both proliferation and differentiation of hematopoietic cells were severely impaired. As a consequence, essentially all lineages were reduced in the periphery. Moreover, the loss of Ash2l resulted in a substantial reduction of hematopoietic cells in BM and spleen. However, LSK cells, which are enriched for hematopoietic stem and progenitor cells, strongly increased in the BM. The loss of Ash2l was paralleled by reduced H3K4 methylation and altered gene expression. In particular, genes associated with G2/M cell cycle progression were prominently down-regulated. We suggest that the inability to progress efficiently through the cell cycle, in particular through the G2-phase, results in the accumulation of LSK cells. It is possible that these cells are particularly sensitive to the altered regulation of genes associated with G2/M and as a consequence are unable to proliferate and differentiate, explaining the reduced MP population as well as further differentiated cells. Of note, others have reported effects on proliferation when components of KMT2 complexes have been manipulated. This is particularly evident for studies addressing MLL1 and WDR5^62–66^.

Biochemical studies have shown that ASH2L, as well as the other WRAD subunits WDR5, RBBP5 and DPY30, is important for KMT2 catalytic activity^10,11,49,67^, and the knockdown of *ASH2L* in cells results in reduced methylation of H3K4^41,67,68^. Similar effects have been reported upon knockdown of other WRAD components^67,69–71^. Thus, the WRAD complex is necessary for efficient H3K4 methylation both *in vitro* and in cells. The KO studies on *Ash2l*, as shown here, and on *Dpy30*, now extend these results to the mouse and provide *in vivo* evidence for the role of these WRAD components^26,28^. These models will allow a more in-depth molecular evaluation of these proteins.

The consequences of deleting *Dpy30* have been analyzed in mice. *Dpy30* KO embryos die early after implantation^29^, and thus reach a more mature developmental stage compared to *Ash2l* KO embryos^25^. In a recent study, *Dpy30* was deleted in the hematopoietic system using Mx1-Cre^28^. The phenotypic consequences are similar compared to our *Ash2l* KO. The KO of either *Ash2l* or *Dpy30* resulted in an exhaustion of peripheral hematopoietic cells, including RBCs, platelets, and WBCs. In parallel, a decrease in the cellularity of the BM was noticed, which was more pronounced in *Ash2l* compared to *Dpy30* KO mice, also when considering that in our study the analysis of the phenotype was performed one day earlier (Figs 1 and 2)^28^. In both models the number of LSK cells in the BM increased, in the case of the *Ash2l* KO almost 20-fold. Further fractionation revealed a specific accumulation of MPP cells, which represent more differentiated LSK cells. Again the differences appeared more pronounced in the *Ash2l* KO than in the *Dpy30* KO (Fig. 3)^28^. Together, these findings suggest that both Ash2l and Dpy30 are necessary for early hematopoiesis and that in the absence of either protein differentiation beyond the MPP state is abrogated.

The LSK cells in *Ash2l* KO animals accumulated in the G2/M-phase of the cell cycle and stopped proliferating (Fig. 5). Of note, we did not observe an increase in mitotic cells, indicating that the LSK cells were enriched in the G2-phase. These effects are compatible with the gene expression analysis (Fig. 4). Essentially all biological processes with high significance in the GO analysis of down-regulated genes in the *Ash2l* KO animals were associated with late cell cycle processes and with the G2/M-phase transition (Fig. 4c and Table S4). Among the genes we identified were many that encode key factors, including Plk1, Aurora A kinase, cyclin B1, and Cdk1, controlling the G2/M transition and thus are required for cells to enter mitosis^72,73^. Moreover, *Mybl2*, which encodes B-Myb, is down-regulated in *Ash2l* KO LSK cells. B-Myb expression and function is associated with proliferating cells and more specifically with promoting the progression through the G2-phase and preparing cells for the transition into mitosis^74^. B-Myb has been reported to activate the above-mentioned G2/M-phase genes^75^. Consistent with these findings, the loss of B-Myb in the hematopoietic system interferes with proliferation and differentiation of stem and progenitor cells, albeit with substantial delay compared to the *Ash2l* KO^76^. This strong association with genes encoding late cell cycle regulators was unexpected, suggesting that these genes are particularly sensitive to a loss of Ash2l and subsequent H3K4 methylation. High expression of ASH2L correlates with poor prognosis in patients with acute myelogenous leukemia harboring the *Flt3-ITD* oncogene^47^. Also, ASH2L correlates positively with the expression of proliferation-associated genes and inversely with genes encoding cell cycle inhibitors^47^. Thus, these findings suggest that ASH2L may act in an oncogenic fashion by directly or indirectly upregulating a gene program associated with proliferation and cell cycle progression.

We observed a rapid decrease in cellularity of the BM and the spleen (Fig. 1). The lack of proliferation in response to the loss of Ash2l is most likely contributing to this observation. In addition, we observed less mature granulocytes (Fig. 1d,f), with aberrant mitosis (Fig. 5f), but increased size and granularity (FSC/SSC; Figs 1l and 3a). This was unexpected indicating that less mature, banded granulocytes as determined in histological sections appeared more mature as judged from their increased SSC in flow cytometry. This seems to reflect an Ash2l-specific phenotype.

In addition to the loss of proliferation, we suspected other relevant functional processes, including induction of apoptosis, to contribute to the observed cell loss in the BM. However, we did not measure a strong apoptotic response (Fig. 6). Consistently, we also did not observe a strong increase in debris-loaded macrophages that would be responsible to remove the remains of dead cells. We expect that during the treatment many hematopoietic cells will leave the BM. Thus, the decrease in cellularity of more than 50% is likely the consequence of a block of proliferation combined with an emigration of mature cells that have managed to differentiate sufficiently. It is also possible that the cells become less adherent as a consequence of altered gene expression that facilitates migration out of the BM.

Compared to the strong G2/M arrest in the *Ash2l* KO cells (Fig. 5), no obvious specific cell cycle arrest was reported in *Dpy30* depleted LSK cells^28^. The KO of *Mll1* also reduces LSK cell proliferation and interferes with hematopoietic stem cell self-renewal^19,20,77^. This is notable because of the different KMT2 enzymes, MLL1 has been suggested to be especially relevant in hematopoietic stem and progenitor cells^30^. Using Mx1-Cre, the KO of *Mll1* resulted in severe BM cytopenia by day 19, suggesting that other KMT2 proteins might compensate for the loss of Mll1^19^. Thus, consistent with a key role of H3K4 methyltransferase complexes in controlling timely gene expression, all three components of KMT2 complexes, Ash2l, Dpy30, and Mll1, are essential for adult hematopoiesis. In all three KO models many genes were repressed, but also a considerable number of genes were activated (Fig. 4 and Table S5)^28,77^, suggesting many indirect effects. Of note is that the gene expression analyses in *Mll1* KO or *Dpy30* KO LSK cells did not reveal a comparably strong G2/M-phase signature as seen in the *Ash2l* KO, consistent with the above discussed phenotypes (Fig. 4 and Table S4)^28,77^. This may well reflect that Mll1 is complemented by other KMT2 enzymes and that Ash2l and Dpy30 have distinct functions in controlling catalytic activity and/or in targeting KMT2 complexes to target genes^10^. This aspect will be interesting to study in more detail.

Mature hematopoietic cells participate in controlling stem and progenitor cells by preventing their proliferation and differentiation^58^. For example, BM megakaryocytes, which decrease upon Ash2l loss (Fig. 2), produce the chemokine CXCL4 and thereby interfere with HSC proliferation^78^. Moreover, platelets and myeloid cells, which are reduced in the *Ash2l* KO animals (Fig. 2), directly and indirectly regulate HSC proliferation and differentiation^79–83^. The substantial decrease in mature cells, and thus the lack of negative feedback signals, is likely to contribute to the accumulation of LSK cells. However, their further maturation is blocked as the loss of Ash2l prevents LSK cells from progressing through mitosis.

Because of the similarities between the findings obtained in the *Ash2l* and *Dpy30* KO models, the observations suggest strongly that deregulation of H3K4 methylation is an important downstream effect in both. Nevertheless, we cannot exclude that at least part of the observed phenotypes is due to other functions of Ash2l and/or Dpy30. The analysis of different WRAD complex components using label-free quantitative mass spectrometry suggested that all four subunits are expressed, at least in HeLa cells, at higher levels than the 6 enzymatic KMT2 subunits, which is particularly evident for WDR5 and DPY30^24^. Indeed, for both WDR5 and DPY30 KMT2 complex independent functions have been reported. WDR5 has been described to promote DNA binding of the oncoprotein MYC^84^. Both WDR5 and DPY30 have been found to be involved in nucleosome remodeling by interacting with the NuRD and the NURF complexes, respectively^85,86^. It seems possible that Ash2l also performs additional molecular functions beyond those associated with H3K4 methylation, however, this remains to be discovered^10^. Thus, the KO of *Ash2l* or *Dpy30* might have additional consequences that are not related to KMT2 complex activities. Despite these uncertainties, the similarities of the phenotypes discussed above argue that the primary effects are due to dysregulated KMT2 complex functions.

The H3K4me3 mark correlates tightly with open, accessible promoters and several possible mechanisms have been described to explain its functional relevance, including the recruitment of TFIID^10,87^. Therefore, inhibition of gene transcription was expected as a response to a decrease in H3K4me3. However, we also observed that many genes were up-regulated, a finding which awaits explanation. Because many genes are down-regulated that participate in chromatin organization (Table S4), the up-regulation of gene expression is likely a secondary effect. The analysis of the promoters of the up-regulated genes did not reveal obvious common transcription factor binding sites (data not shown). Further studies will be required to address the intricate mechanisms underlying this complex network of Ash2l regulated gene expression.

## Materials and Methods

### Animals

Mice used in this study were bred in the in-house, specific pathogen-free (SPF) animal facility (Institute for Laboratory Animal Science, Medical Faculty, RWTH Aachen University) and maintained on a 12 h light/dark cycle in cages with up to five mice and food and water provided *ad libitum*. All procedures were approved by the Institute for Laboratory Animal Science and the local government (LANUV NRW, AZ 84-02.04.2013.A182) in accordance with German and EU regulations.

To generate mice with the possibility to conditionally knockout Ash2l protein expression, ES cells were obtained from the EUCOMM gene targeting program (ID 35610)^88^, harboring a loxP-flanked exon 4 of *Ash2l* as part of a targeting cassette. The albino mouse strain B6N-Tyr^c-Brd^/BrdCrCrl was employed to generate blastocysts for microinjection. Chimeric offspring were bred to assess germ line transmission. Mice harboring an improved *FlpO* transgene (C57BL/6N Tg(CAG-Flpo)1Afst) were then used for the deletion of the targeting cassette^26^. For the conditional removal of the floxed exon 4 of *Ash2l*, a mouse strain with a transgene expressing Cre recombinase protein under control of the Mx1 promoter (B6.Cg-Tg(Mx1-cre)1Cgn/J) was obtained. Littermates were crossed to generate the conditional KO genotype *Ash2lfl*/*flTg*(*Mx1*-*Cre*) and different control animals (*Ash2lwt*/*wt*, *Ash2lwt*/*wtTg*(*Mx1*-*Cre*), *Ash2lwt*/*fl*, *Ash2lwt*/*flTg*(*Mx1*-*Cre*), *Ash2lfl*/*fl*) without or with a single copy of the *Cre* transgene. For the induction of Cre recombinase expression, animals at the age between 10 and 25 weeks were injected intraperitoneally (ip) with synthetic double stranded RNA poly(I:C) (pIC; 10 mg/kg body weight) three times at alternating days (for details of chemicals and antibodies see Table S1). The analyses were performed at day ten following the first injection (Fig. 1a) (see below). Among the control animals and animals of different age and sex no differences in their response to the pIC treatment could be observed.

### DNA analyses

The Cre- and Ash2l-specific genotypes were determined from DNA of tail biopsies using primer pairs Cre_for_/Cre_rev_ and P1/P2, respectively (Table S2 and Fig. 1a). The knockout efficiency of exon 4 of *Ash2l* after treatment of the mice with pIC was assessed using genomic DNA of bone marrow (BM) cells isolated with the High Pure PCR Template Preparation Kit and primer pair P3/P4 (Table S2). The GoTaq Green Master Mix or the 5xPCR Master Mix were used for the end-point PCR reactions.

### RNA analyses

For gene transcription analyses RNA was prepared with the RNeasy Mini or Micro Kits, depending on the number of cells available, and cDNA was generated with the QuantiTect Reverse Transcription Kit. SYBR Green kits were employed for the real time PCR analyses (SensiMix Sybr No-Rox kit, SensiFast Sybr No-Rox kit, QuantiNova SYBR Green PCR kit) and performed in a RotorGene 6000 (Corbett, Qiagen). QuantiTect Primer Assays or primers designed with the Primer3web software were used as indicated (Table S3). The efficiency of all primers used was higher than 95%. The PCR reactions were performed for 30 cycles at 95 °C for 15 s, 60 °C for 15 s, and 72 °C for 15 s. Fold changes in expression were calculated according to Pfaffl^89^;

$${\rm{relative}}\,{\rm{expression}}=\frac{{{{\rm{E}}}_{{\rm{target}}}}^{{\rm{\Delta }}C{P}_{{\rm{target}}}(\mathrm{control}-\mathrm{sample})}}{{{{\rm{E}}}_{{\rm{reference}}}}^{{\rm{\Delta }}C{P}_{{\rm{reference}}}(\mathrm{control}-\mathrm{sample})}}),$$

measuring the crossing point CP and taking into account the respective reaction efficiencies E, using ß-glucuronidase (*Gusb*) as the reference gene as its expression was unaffected by the pIC treatment of the mice regardless of their genotype.

### Western blots

Cells were lysed on ice in RIPA buffer (10 mM Tris/HCl, pH 7.4, 150 mM NaCl, 1% Nonidet P-40, 1% deoxycholic acid, 0.1% SDS) containing a protease inhibitor cocktail and 10 mM sodium butyrate. The lysates were sonicated for 15 min with 30 seconds on/off cycles in the cold and centrifuged (10,000 × g, 5 min, 4 °C). From the supernatants the protein concentration was measured and 30 µg of total proteins were used. For western blots of LSK sorted cells (see below), the flow-through from the RNeasy Micro Kit column of 1.5 × 10^6^ cells was incubated with 4 volumes of ice-cold acetone (30 min, on ice) and centrifuged (13.000 × g, 10 min, 4 °C). The pellets were washed with ice-cold ethanol (100%), air dried and taken-up in sample buffer. For detection the antibodies in 5% low-fat milk, 0,05% Tween20 in PBS (anti-ASH2L 1:2000, anti-actin 1:200, and peroxidase anti-rabbit and -mouse 1:2000) together with the SuperSignal Femto Substrate were used.

### Immunohistochemical analysis

For immunohistochemistry (IHC) sternum bones were fixed in 4% formaldehyde, decalcified in 10% EDTA (pH 7.4), embedded in paraffin and cut into 2 µm sections. Following deparaffinization in xylene (3 × 5 min) and rehydration in decreasing ethanol concentrations (100%, 95%, 70%; 3 min each) and finally placed in water, serial sections were immersed in target retrieval solution (pH 6) at 95 °C for 20 min. For all washing steps (3 × 5 min) wash buffer (1x) was used. Following the incubation with peroxidase-blocking solution, the sections were treated with 2.5% normal horse serum for 30 min and incubated at room temperature for 30 min with the primary antibodies (1:750) in antibody diluent. A control staining with isotype Ig was performed on the same slide on a second tissue section with identical staining procedures. For detection the ImmPress peroxidase kit was used (20 min), followed by incubation with DAB (10 min). Sections were counterstained with Mayer’s hematoxylin. For the chloroacetate esterase stain, slides were incubated to ASDCL solution for 80 min (ASDCL-solution: Hexazonium-solution (HCl, parafuchsine hydrochlorid, sodium nitrit in PBS buffer) and naphtol-AS-D-choloracetate esterase in DMSO buffer). After counterstain, mounting was performed with glycerol gelatin. All other stains were performed after identical depraffination und rehydration as well as dehydration. After dehydration they were mounted, scanned with the NanoZoomer from Hamamatsu Photonics and visualized using the NDP.view2 software. High magnification photographs were taken by a Zeiss microscope with a 100x oil objective (Zeiss, Jena, Germany) and an Olympus camera and software (Analysis 5.1, Olympus, Hamburg, Germany).

Cell counts were performed using a widely used standard in diagnostics, the so called high-power-field (HPF) analysis, indicating the visible area in 400x magnification (40x objective). The diameter of HPF ranges roughly from about 0.45 mm to 0.65 mm with a mean of 0.55 mm and an area of about 0.24 mm^2^.

### Survival

For survival analysis mice were assessed using body condition scoring. Reaching the endpoint, moribund mice were euthanized and the survival times documented using a Kaplan-Meier plot.

### Blood analyses

After anesthesia with isoflurane, blood taken from the retro-orbital sinus was collected and an aliquot was mixed immediately with EDTA for a full blood exam. WBC: white blood cells, PLT (platelets), RBC (red blood cells), HGB (hemoglobin), HCT (hematocrit), MCV (mean corpuscular volume), MCH (mean corpuscular hemoglobin), MCHC (mean corpuscular hemoglobin concentration). Measurements were performed by the Laboratory of Clinical Chemistry and Hematology, Institute for Laboratory Animal Science, Medical Faculty, RWTH Aachen University.

### Flow cytometry analysis of fixed cells

Femurs and tibias were flushed with PBS containing 2% fetal calf serum (FCS) and the BM cells were passed through a 70 µm filter and counted (BM refers to the cells flushed from 2 tibias and 2 femurs/mouse). Aliquots of 10^6^ cells were centrifuged (400 × g, 5 min, RT), taken up in 1 ml Fixation/Permeabilization buffer (1x) of the FoxP3 Transcription Factor Staining Buffer Kit and incubated for 30 min at RT. After centrifugation as above, the cells were dispersed in 2 ml Permeabilization buffer (1x) of the same kit and centrifuged again. After pouring off the supernatant, 20 µl of the anti-H3 antibody (1:400 in 1x Permeabilization Buffer, 40 min, RT) was added. For washing 2 ml Permeabilization Buffer was added and the cells were centrifuged and stained as above using anti-rabbit-AlexaFluor633 as secondary antibody. For double stainings of the cells, the second primary antibody was added and incubated and washed as above. For detection anti-rabbit-AlexaFluor488 antibody was used as described above. For evaluation the cells were taken up in 2% horse serum in PBS. Flow cytometry measurements were performed in a FACSCANTO II system from BD Biosciences equipped with FACSDIVA V6.1.3 software. For analyses FlowJo software was employed. To assess the respective staining intensities the main population of cells was selected by gating within the FSC/SSC plot.

### Flow cytometry analysis of surface markers

Bone marrow cells were flushed with PBS/2% FCS from femurs and tibias and treated as above. Spleen tissue was minced and squeezed through a 100 µm cell strainer to obtain single cell suspensions. After lysis of erythrocytes with 3 successive incubations (2 min, RT) in AKC-lysis buffer (0.15 M NH_4_Cl, 1 mM KHCO_3_, 0.1 mM Na_2_EDTA, pH 7,3), cells were washed with PBS/2% FCS and live cells were counted with a CASY Cell Counter (Omni Life Science). Per 1 × 10^6^ cells 0.5 µl of the respective antibody was used, except for CD48, CD117, CD16/32, CD150 and Sca-1 (1 µl per 1 × 10^6^ cells) or CD34 (2 µl per 1 × 10^6^ cells). Incubation time was 15 min at RT, except for CD34 (1.5 h at 4 °C). The biotin-labelled Sca-1 antibody was stained with Streptavidin-PE-Cy7 for 15 min. For progenitor cell analysis, CD34 staining was done first followed by the addition of further antibodies. The gating strategy is outlined in Fig. 2a. Flow cytometry measurements were conducted with a FACS Gallios system (Beckman Coulter) and analyzed with FlowJo or Kaluza Software.

### LSK cell sorting

BM cells were prepared as described above followed by lineage depletion using the MACS Lineage Cell Depletion Kit. Lineage negative (lin^−^) cells were stained with Sca-1 and CD117 (c-Kit) antibodies and the +/+cell population (LSK) was gated for cell sorting performed on a FACSAria I cytometer (BD Biosciences).

### Proliferation assay

LSK cells (25’000) were seeded in triplicates in 100 µl StemSpan^TM^ medium containing IL-3 (20 ng/ml), IL-6 (20 ng/ml), SCF (50 ng/ml) and 20% BIT 9500 serum substitute. Fresh medium was added every two days and the cells were counted at the indicated days and a growth curve was computed.

### Colony forming assay

LSK cells (1000 cells/ml) were seeded in triplicates in methylcellulose (MethoCult GF) and colonies were counted seven days after seeding using a light microscope.

### Cell cycle analysis using DAPI staining

BM cells were centrifuged, taken up in 500 µl PBS and fixed at −20 °C by adding 2 ml cold methanol. After washing the cells in PBS, they were treated with RNaseA (20 µg/ml) for 5 min, washed, stained with DAPI (6.7 µg/ml) followed immediately by flow cytometry measurement and analysis with the FlowJo software.

### Cell cycle analysis using BrdU incorporation

Mice were injected (i.p.) with BrdU (100 mg/kg body weight) 2 h before preparation of the cells. LSK cells were purified as described above and 2 × 10^5^ cells (from a pool of 3 control mice and of 3 individual KO mice) were taken up in 100 µl PBS/2% FCS and mixed with 1× BrdU staining buffer (1 ml, BrdU Staining Kit FITC) and incubated for 20 min (RT, in the dark). Cells were washed with 1 ml PBS/2% FCS, centrifuged (700 × g, 5 min) and resuspended in 100 µl DNAse I working solution, followed by incubation for 1 h at 37 °C in the dark. Subsequently cells were washed twice with PBS/2% FCS, centrifuged (700 × g, 5 min) and 5 µl anti-BrdU antibody in 100 µl PBS/2% FCS was added, followed by 30 min incubation at RT in the dark. Then the cells were washed twice, 5 µl 7-AAD in 100 µl PBS/2% FCS was added and incubated for 1 min before flow cytometry analysis with a FACS Gallios (Beckman Coulter) and analyzed with the FlowJo software.

### Apoptosis measurement

For the assessment of apoptotic cells, isolated BM cells were applied to MACS lineage depletion as for cell sorting (see above) and 3 × 10^5^ lin^−^ cells were seeded in 300 µl StemSpan^TM^ medium supplemented with each 20 ng/ml IL-3 and IL-6 and 50 ng/ml SCF. After 48 and 72 h, apoptosis was measured using the Pacific Blue Annexin V Apoptosis Detection Kit with 7-AAD. Flow cytometry measurements were performed with the FACS Gallios system (Beckman Coulter) and analyzed with FlowJo software taking together the values of both time points, as they did not differ. Apoptotic cells in histological sections were identified using TdT-mediated dUTP-biotin nick end labeling (TUNEL) according to the specifications of the manufacturer (Promega).

### Complementation of *Ash2l* knockout

To generate viral particles for the infection of bone marrow cells, the lentiviral vector pLeGO-iT2-Puro+^90^ without (for empty virus, EV) or with the cDNA for human ASH2L (for hASH2L virus) was used. Together with the helper plasmids pMDLg/p RRE and pRSV-Rev^91^ and the envelope plasmid pLP/VSVG they were transfected into HEK293T cells by calcium phosphate precipitation. After 24 h the precipitate was washed away and the cells were refed. After a further 24 and 48 h the medium was collected and filtrated through a 0.45 µm cellulose acetate filter. Non-coated 6-well plates were treated successively with Retronectin (1:12.5 in PBS, 4 °C, overnight) and 2% human serum albumin (RT, 30 min). After washing with 1x HBSS the virus-containing medium was added in two successive rounds.

Lin^−^ cells (as for LSK cell sorting, see above) were grown for 48 h in IMDM medium supplemented with IL-3 and IL-6 (each 20 ng/ml), SCF (50 ng/ml) and 20% BIT 9500 serum substitute and then added to the virus-coated wells. After 48 h 4000 cells of knockout mice and 2000 cells of wt mice (transduced with EV vs. hASH2L virus, respectively) were seeded per well in 1 ml MethoCult GF (triplicates, 3 mice each) and colonies were counted after 7 days.

### Microarray

LSK cells were prepared (as for LSK cell sorting, see above), the RNA was isolated with the RNeasy Micro Kit and the quantity and quality was assessed using NanoDrop (Thermo Fisher Scientific) and Agilent Bioanalyzer (Agilent Technologies). Fourty ng RNA (RNA integrity number RIN >9) per sample was transcribed and labeled with the Ovation Pico WTA System v2 and the Encore Biotin Module (both Nugen). The resulting fragmented and biotinylated cDNA was hybridized to MTA-1.0 arrays and visualized using the Affymetrix GeneChip Fluidics Station 450 with the Affymetrix wash and stain kit (45 °C, 60 rpm, 18 h = hybridization conditions, FS450 0001 = wash and stain program). The data are uploaded as described in Table S1.

### Bioinformatics

Data were normalized with the Signal Space Transformation (SST) in conjunction with robust multiple-array (RMA) average normalization method (SST-RMA) (Affymetrix, Microarray normalization using Signal Space Transformation with probe Guanine Cytosine Count Correction). Differential gene expression was performed with Limma with a corrected p-value below 0.05. These genes were provided as input for a gene ontology (GO) enrichment analysis with g:profiler. Next, we used REVIGO to display the association between all enriched GO terms (p-value < 0.00001) from the biological process ontology.

### Quantification and statistical analysis

Error bars represent standard deviation (SD) of the mean, unless otherwise indicated. Statistical significance was evaluated by an unpaired, two-tailed Student’s t test using GraphPadPrism software, unless otherwise indicated (****p < 0.0001; ***p < 0.001; **p < 0.01; *p < 0.05).

### Ethical approval and informed consent

All procedures were approved by the Institute for Laboratory Animal Science and the local government (LANUV NRW, AZ 84-02.04.2013.A182) in accordance with German and EU regulations (also indicated in Material and Methods section).

### Gene expression analysis

GSE114433 (also indicated in Table S1).

## Supplementary information

Supplementary information
