# Supplementary material for: Hematopoietic stem and progenitor cell proliferation and differentiation requires the trithorax protein Ash2l
Source: Sci Rep. 2019 Jun 4;9:8262. doi: 10.1038/s41598-019-44720-3 (PMC6547667; doi:10.1038/s41598-019-44720-3)
Supplement: Supplementary file 1 — Supplementary information [file 41598_2019_44720_MOESM1_ESM.pdf]

SUPPLEMENTARY INFORMATION

Hematopoietic stem and progenitor cell proliferation and differentiation requires the trithorax protein Ash2l

Juliane Lüscher-Firzlaff[1,*], Nicolas Chatain[2,*], Chao-Chung Kuo[3], Till Braunschweig[4], Agnieszka Bochyńska[1], Andrea Ullius[1,†], Bernd Denecke[5], Ivan Costa[3], Steffen Koschmieder[2,‡], and Bernhard Lüscher[1,‡]

[1]Institute of Biochemistry and Molecular Biology, [2]Department of Hematology, Oncology, Hemostaseology, and Stem Cell Transplantation, [3]Institute for Computational Genomics, [4]Institute of Pathology, [5]Interdisciplinary Center for Clinical Research Aachen, Faculty of Medicine, RWTH Aachen University, Pauwelsstrasse 30, 52074 Aachen, Germany

[†]Present address: QIAGEN GmbH, Qiagen Str.1, 40724 Hilden, Germany

[*]Contributed equally

[‡]Equally contributing senior authors, correspondence to S.K. (email: skoschmieder@ukaachen.de) and B.L. (luescher@rwth-aachen.de)

Table S1, key resources

Table S2, Primers for endpoint PCR

Table S3, Primers for RT-qPCR

Table S4, GO terms of down-regulated genes

Table S5, GO terms of up-regulated genes

Table S1

KEY RESOURCES TABLE

REAGENT or RESOURCE	SOURCE	IDENTIFIER
Antibodies		
Rabbit monoclonal anti-ASH2L (D93F6)	Cell Signaling Technology	5019S, RRID:AB_1950350
Mouse monoclonal anti-actin (C4)	MP Biomedicals	08691001, RRID:AB_2336056
Rabbit monoclonal anti-Ki67	Thermo Fisher Scientific	RM-9106-S1, RRID:AB_2341197
Rabbit monoclonal anti-F4/80	Abcam	ab6640, RRID:AB_1140040
Rabbit polyclonal anti-Phospho-Histone H3 (Ser10)	Cell Signaling	9701, RRID:AB_331535
Rat anti-CD3 Monoclonal Antibody, PE-Cy5 Conjugated (Clone 17A2)	BD Biosciences	Cat# 555276, RRID:AB_395700
Rat anti-mouse CD3 Monoclonal Antibody, Pacific Blue Conjugated	BioLegend	Cat# 100214, RRID:AB_493645
Rat anti-CD4 Monoclonal Antibody, PE-Cy5 Conjugated (Clone RM4-5)	BD Biosciences	Cat# 553050, RRID:AB_394586
Rat anti-mouse CD4 Monoclonal Antibody, PE/Cy7 Conjugated (Clone RM4-5)	BioLegend	Cat# 100528, RRID:AB_312729
Rat anti-mouse CD8a Monoclonal Antibody, PE/Cy5 Conjugated (Clone 53-6.7)	BioLegend	Cat# 100710, RRID:AB_312749
Rat anti-mouse CD8a Monoclonal Antibody, APC Conjugated (Clone 53-6.7)	BioLegend	Cat# 100712, RRID:AB_312751
Rat anti-mouse/human CD11b Monoclonal Antibody, PE/Cy5 Conjugated (Clone M1/70)	BioLegend	Cat# 101210, RRID:AB_312793
Rat anti-mouse/human CD11b Monoclonal Antibody, PE/Cy7 Conjugated (Clone M1/70)	BioLegend	Cat# 101216, RRID:AB_312799
Rat anti-mouse CD16/CD32 Monoclonal Antibody, APC Conjugated (Clone 93)	Thermo Fisher Scientific	Cat# 17-0161-81, RRID:AB_469355
Rat anti-mouse CD34 Monoclonal Antibody, eFluor 450 Conjugated (Clone RAM34)	Thermo Fisher Scientific	Cat# 48-0341-82, RRID:AB_2043837
Rat anti-mouse CD41a Monoclonal Antibody, APC-eFluor 780 Conjugated (Clone MWReg30)	Thermo Fisher Scientific	Cat# 47-0411-82, RRID:AB_2573958
anti-mouse CD45.1 Monoclonal Antibody, Phycoerythrin Conjugated (Clone A20)	BD Biosciences	Cat# 553776, RRID:AB_395044
Rat anti-mouse/human CD45R/B220 Monoclonal Antibody, PE/Cy5 Conjugated (RA3-6B2)	BioLegend	Cat# 103210, RRID:AB_312995

Hamster anti-mouse CD48 Monoclonal Antibody, FITC Conjugated (HM48-1)	Biolegend	Cat# 103404, RRID:AB_313019
Rat anti-mouse CD117/c-kit Monoclonal Antibody, APC / Cy7 Conjugated (Clone 2B8)	Biolegend	Cat# 105826, RRID:AB_1626278
Rat anti-mouse CD150 (SLAM) Monoclonal Antibody, APC Conjugated (Clone TC15-12F12.2)	Biolegend	Cat# 115910, RRID:AB_493460
Rabbit polyclonal anti-H3K4me3	Abcam	ab8580, RRID:AB_306649
Rabbit polyclonal anti-H3K4me1	Abcam	ab8895, RRID:AB_306847
Rabbit polyclonal anti-H3	Abcam	ab1791, RRID:AB_302613
Rabbit polyclonal anti-H3K9ac	Abcam	ab10812, RRID:AB_297491
Peroxidase-AffiniPure Goat Anti-Rabbit IgG (H+L)	Jackson Immuno Research Labs	111-035-144, RRID:AB_2307391
Peroxidase-AffiniPure Rat Anti-Mouse IgG (H+L)	Jackson Immuno Research Labs	415-035-166, RRID:AB_2340269
Goat anti-Rabbit IgG (H+L) Cross-Adsorbed Secondary Antibody, Alexa Fluor 488	Molecular Probes	A11008, RRID:AB_143165
Goat anti-Rabbit IgG (H+L) Cross-Adsorbed Secondary Antibody, Alexa Fluor 633	Molecular Probes	A21070, RRID:AB_2535731
Rat anti-mouse Ly-6G/Ly-6C (Gr-1) Monoclonal Antibody, PE/Cy5 Conjugated (RB6-8C5)	Biolegend	Cat# 108410, RRID:AB_313374
Rat anti-mouse Ly-6G/Ly-6C (Gr-1) Monoclonal Antibody, FITC Conjugated (Clone RB6-8C5)	Biolegend	Cat# 108405, RRID:AB_313370
Streptavidin-Phycoerythrin-Cy7 antibody	BD Biosciences	Cat# 557598, RRID:AB_10049577
Rat anti-mouse TER-119/Erythroid Cells Monoclonal Antibody, PE/Cy5 Conjugated (Clone TER-119)	Biolegend	Cat# 116210, RRID:AB_313711
Rat anti-mouse TER-119/Erythroid Cells Monoclonal Antibody, APC Conjugated (Clone TER-119)	Biolegend	Cat# 116211, RRID:AB_313712
ImmPress Peroxidase Kit, anti-rabbit	Vector Laboratorries	MP-7401, RRID:AB_2336529
ImmPress Peroxidase Kit, anti-rat	Vector Laboratorries	MP-7404, RRID:AB_2336531
**Chemicals, Peptides, and Recombinant Proteins**		
pIC: Poly(I:C)	InvivoGen	tlrl-pic
Protease inhibitor cocktail	Sigma-Aldrich	P8340
Sodium butyrate	Sigma-Aldrich	303410

SuperSignal™ West Femto Maximum Sensitivity Substrate	ThermoFisher Scientific	34095
Target retrieval solution, citrate, pH 6	Dako	S2369
Peroxidase blocking solution	Dako	S2023
Wash Buffer, 10x	Dako	S3006
Antibody diluent	Dako	S2022
DAB: diaminobenzidine	Dako	K3468
DAB ImmPact	Vector Laboratories	SK-4105, RRID:AB_2336520
Mayer's hematoxylin	Dako	S3309
Stem Span SFEM	Stemcell Technologies	09600
Murine rec. IL-3	Immunotools	12340033
Murine rec. IL-6	Immunotools	12340063
Murine rec. SCF	Immunotools	12343325
MethoCult GF	Stemcell Technologies	M3434
IMDM	ThermoFisher Scientific	12440053
FCS: Fetal bovine serum (Gibco)	ThermoFisher Scientific	10500064
BIT 9500 serum substitute	Stemcell Technologies	09500
Polybrene: hexadimethrine bromide	Sigma-Aldrich	H9268
DAPI: 4′,6-Diamidine-2′-phenylindole dihydrochloride (Roche)	Sigma-Aldrich	10236276001
BrdU: 5-Bromo-2′-deoxyuridine (Roche)	Sigma-Aldrich	10280879001
Retronectin	Takara	T100B
HBSS (Gibco)	ThermoFisher Scientific	14025092
Critical Commercial Assays		
High Pure PCR Template Preparation Kit	Roche	11 796 828 001
RNeasy Mini Kit	Qiagen	74106
RNeasy Micro Kit	Qiagen	74004
QuantiTect Reverse Transcription Kit	Qiagen	205313
Go Taq® Green Master Mix	Promega	M7123
5xPCR Master Mix	BioSell	91.731.1000
QuantiNova SYBR Green PCR Kit	Qiagen	208056
SensiMix Sybr No-Rox Kit	Bioline	QT650-20
SensiFast Sybr No-Rox Kit	Bioline	98020
Foxp3 / Transcription Factor Staining Buffer Set	eBioscience	00-5523
BrdU Staining Kit for Flow Cytometry FITC	eBioscience	8811-6600
DeadEnd Colorimetric TUNEL System	Promega	G7130
MACS Lineage Cell Depletion Kit (mouse)	Miltenyi Biotec	130-090-858
Pacific Blue Annexin V Apoptosis Detection Kit, 7-AAD	Biolegend	640926

Ovation Pico WTA System v2	Nugen	3302
Encore Biotin Module	Nugen	4200
Applied Biosystems GeneChip wash and stain kit	ThermoFisher Scientific	900720
Deposited Data		
GEO repository	https://www.ncbi.nlm.nih.gov/geo/	GSE114433 Token: itylsieydtwblmj
Experimental Models: Cell Lines		
ES cells: C57BL/6N Ash2l$^{tm1a(EUCOMM)Wtsi}$	EuMMCR	IKMC project 35610
Experimental Models: Organisms/Strains		
Mouse: C57BL/6N-*Tyr*$^{c-Brd}$/BrdCrCrl	Charles River	RRID:IMSR_CRL:493
Mouse: C57BL/6N Tg(CAG-Flpo)1Afst	Ref [1]	RRID:MMRRC_036512-UCD
Mouse: B6.Cg-Tg(Mx1-cre)1Cgn/J	Ref [2]	RRID:IMSR_JAX:003556
Oligonucleotides		
Table S3: Primers for endpoint PCR	This paper	N/A
Table S4: Primers for RT-PCR	This paper	N/A
Recombinant DNA		
pLeGO-iT2-Puro+	Ref [3]	
Plasmid pMDLg/p RRE	Addgene	12251
Plasmid pRSV-Rev	Addgene	12253
Plasmid pLP/VSVG	Invitrogen	K4975-00
Software and Algorithms		
Primer3web	http://primer3.ut.ee	4.1.0
Prism6	Graphpad	6.0h for MacOSX
NDP.view	Hamamatsu Photonics	2
FACSDIVA	BD Biosciences	6.1.3
Kaluza	Beckman Coulter	1.3
FlowJo	BD Biosciences	10.2
Expression Console software	ThermoFisher Scientific	TAC software 4.0.1
Limma	https://bioconductor.org/packages/release/bioc/html/limma.html	3.7
g:profiler	https://biit.cs.ut.ee/gprofiler/	Version r1741_e90_eg37
REVIGO	http://revigo.irb.hr/	1.0
Other		

GeneChip Mouse Transcriptome Assay	Affymetrix	MTA-1

References

1 Kranz, A. *et al.* An improved Flp deleter mouse in C57Bl/6 based on Flpo recombinase. *Genesis* **48**, 512-520, doi:10.1002/dvg.20641 (2010).

2 Kuhn, R., Schwenk, F., Aguet, M. & Rajewsky, K. Inducible gene targeting in mice. *Science* **269**, 1427-1429 (1995).

3 Weber, K., Mock, U., Petrowitz, B., Bartsch, U. & Fehse, B. Lentiviral gene ontology (LeGO) vectors equipped with novel drug-selectable fluorescent proteins: new building blocks for cell marking and multi-gene analysis. *Gene Ther* **17**, 511-520, doi:10.1038/gt.2009.149 (2010).

Table S2

Primers for endpoint PCR		
Cre_{for}: catttgggccagctaaacat	IKMC project 35610	Cre_F
Cre_{rev}: taagcaatccccagaaatgc	IKMC project 35610	Cre_R
P1: catccacaggtcatgtctgc	IKMC project 35610	loxP for
P2: agccgtgatggagtgtaacc	IKMC project 35610	loxP rev
P3: aaggcgcataacgataccac	IKMC project 35610	5FRT-F
P4: actgatggcgagctcagacc	IKMC project 35610	floxed LR

Table S3

Primers for RT-qPCR		
Mm_Ash2l $exon4_{for}$: ccgctgacacctttggaata	This paper	Not applicable
Mm_Ash2l $exon4_{rev}$: ttgcttccggagaaagtagg	This paper	Not applicable
Mm_Car1_2_SG	Qiagen	QT02533139
Mm_Cdh17_1_SG	Qiagen	QT01055110
Mm_Chil3_1_SG	Qiagen	QT00108829
Mm_Cited 2_{for}: catcggctgtccctctatgt	This paper	Not applicable
Mm_Cited 2_{rev}: atggtctgccatttccagtc	This paper	Not applicable
Mm_Dntt_1_SG	Qiagen	QT00112693
Mm_Gusb_1_SG	Qiagen	QT00176715
Mm_$Irf8_{for}$: ggatgtgtgaccggaatggt	This paper	Not applicable
Mm_$Irf8_{rev}$: tgcccaggccttaaaaatgga	This paper	Not applicable
Mm_Mgam_2_SG	Qiagen	QT01750616
Mm_Myc_1_SG	Qiagen	QT00096194
Mm_Plk1_1_SG	Qiagen	QT00112371
Mm_Prg2_1_SG	Qiagen	QT00133007
Mm_$S100a9_{for}$: cagcataaccaccatcatcg	This paper	Not applicable
Mm_$S100a9_{rev}$: aaaggttgccaactgtgctt	This paper	Not applicable
Mm_Serpina3f_1_SG	Qiagen	QT00300482

Table S4. GO terms of down-regulated genes.

term.id	term.name	domain	p-value
GO:0007067	mitotic nuclear division	BP	3,16E-33
GO:1903047	mitotic cell cycle process	BP	4,39E-32
GO:0007049	cell cycle	BP	9,62E-32
GO:0000280	nuclear division	BP	9,68E-32
GO:0022402	cell cycle process	BP	1,2E-31
GO:0048285	organelle fission	BP	2,29E-31
GO:0000278	mitotic cell cycle	BP	7,76E-30
GO:0005694	chromosome	CC	7,72E-29
GO:0007059	chromosome segregation	BP	2,78E-25
GO:0051301	cell division	BP	3,27E-25
GO:0044427	chromosomal part	CC	4,39E-24
GO:0000793	condensed chromosome	CC	9,51E-24
GO:0051276	chromosome organization	BP	4,13E-21
GO:0000779	condensed chromosome, centromeric region	CC	1,75E-20
GO:0098687	chromosomal region	CC	1,55E-19
GO:0000776	kinetochore	CC	1,65E-19
GO:0000775	chromosome, centromeric region	CC	3,39E-19
GO:0000777	condensed chromosome kinetochore	CC	2,81E-18
GO:0005819	spindle	CC	4,07E-17
GO:0098813	nuclear chromosome segregation	BP	2,41E-16
GO:0000226	microtubule cytoskeleton organization	BP	2,61E-14
GO:0015630	microtubule cytoskeleton	CC	8,31E-14
GO:0000228	nuclear chromosome	CC	8,98E-14
GO:0007017	microtubule-based process	BP	1,28E-13
GO:0044815	DNA packaging complex	CC	2,13E-13
GO:0044454	nuclear chromosome part	CC	6,46E-13
GO:0051726	regulation of cell cycle	BP	8,39E-13
GO:0000070	mitotic sister chromatid segregation	BP	4,21E-12
GO:0031109	microtubule polymerization or depolymerization	BP	5,11E-12
GO:0070507	regulation of microtubule cytoskeleton organization	BP	9,53E-12
GO:0000819	sister chromatid segregation	BP	1,13E-11
GO:0000786	nucleosome	CC	2,12E-11
GO:0010564	regulation of cell cycle process	BP	4,19E-11
GO:0000785	chromatin	CC	7,14E-11
GO:0032886	regulation of microtubule-based process	BP	9,56E-11
GO:0008017	microtubule binding	MF	1,22E-10
GO:0044430	cytoskeletal part	CC	1,62E-10
GO:0051302	regulation of cell division	BP	1,78E-10
GO:0000910	cytokinesis	BP	2,38E-10
GO:0006323	DNA packaging	BP	4,1E-10
GO:0005856	cytoskeleton	CC	5,11E-10
GO:0000780	condensed nuclear chromosome, centromeric region	CC	7,35E-10
GO:0005876	spindle microtubule	CC	1,8E-09
GO:0007019	microtubule depolymerization	BP	2,13E-09
GO:0007010	cytoskeleton organization	BP	2,84E-09
GO:0032993	protein-DNA complex	CC	5,19E-09
GO:0071103	DNA conformation change	BP	8,67E-09
GO:0034502	protein localization to chromosome	BP	9,87E-09
GO:0008608	attachment of spindle microtubules to kinetochore	BP	1,17E-08
GO:0031110	regulation of microtubule polymerization or depolymerization	BP	1,18E-08
GO:0006325	chromatin organization	BP	1,42E-08
GO:0030496	midbody	CC	1,79E-08
GO:0072686	mitotic spindle	CC	1,95E-08
GO:0005813	centrosome	CC	2,75E-08
GO:0015631	tubulin binding	MF	0,000000035
GO:0000940	condensed chromosome outer kinetochore	CC	3,55E-08
GO:0051781	positive regulation of cell division	BP	5,07E-08
KEGG:04110	Cell cycle	keg	6,08E-08
GO:0007051	spindle organization	BP	7,34E-08
GO:0005815	microtubule organizing center	CC	8,93E-08

GO:0005874	microtubule	CC	9,54E-08
GO:0051493	regulation of cytoskeleton organization	BP	0,000000149
GO:0007346	regulation of mitotic cell cycle	BP	0,000000152
GO:0071459	protein localization to chromosome, centromeric region	BP	0,000000181
GO:0033043	regulation of organelle organization	BP	0,000000193
GO:0051261	protein depolymerization	BP	0,000000328
GO:0071822	protein complex subunit organization	BP	0,00000036
GO:0065004	protein-DNA complex assembly	BP	0,000000483
GO:0010639	negative regulation of organelle organization	BP	0,000000599
GO:0007026	negative regulation of microtubule depolymerization	BP	0,000000613
GO:0000778	condensed nuclear chromosome kinetochore	CC	0,000000746
GO:0031114	regulation of microtubule depolymerization	BP	0,000000751
GO:0090068	positive regulation of cell cycle process	BP	0,000000813
GO:0000794	condensed nuclear chromosome	CC	0,00000112
GO:0045787	positive regulation of cell cycle	BP	0,00000119
GO:0043624	cellular protein complex disassembly	BP	0,0000012
GO:0044877	macromolecular complex binding	MF	0,00000136
GO:0031111	negative regulation of microtubule polymerization or depolymerization	BP	0,00000149
GO:0061640	cytoskeleton-dependent cytokinesis	BP	0,00000153
GO:0045786	negative regulation of cell cycle	BP	0,00000158
GO:0000281	mitotic cytokinesis	BP	0,00000176
GO:0051494	negative regulation of cytoskeleton organization	BP	0,00000198
GO:0051983	regulation of chromosome segregation	BP	0,00000376
GO:0071824	protein-DNA complex subunit organization	BP	0,00000383
GO:0043241	protein complex disassembly	BP	0,00000478
GO:0007052	mitotic spindle organization	BP	0,00000509
GO:0030097	hemopoiesis	BP	0,00000615
GO:0000922	spindle pole	CC	0,00000938
GO:0032984	macromolecular complex disassembly	BP	0,00000965
GO:0070271	protein complex biogenesis	BP	0,0000123
GO:0006461	protein complex assembly	BP	0,0000123
GO:1901880	negative regulation of protein depolymerization	BP	0,0000142
GO:0043242	negative regulation of protein complex disassembly	BP	0,0000166
GO:0051129	negative regulation of cellular component organization	BP	0,000017
GO:0019899	enzyme binding	MF	0,0000205
KEGG:05322	Systemic lupus erythematosus	keg	0,0000256
GO:0044770	cell cycle phase transition	BP	0,0000263
GO:0048534	hematopoietic or lymphoid organ development	BP	0,0000265
GO:0065003	macromolecular complex assembly	BP	0,0000315
GO:1901879	regulation of protein depolymerization	BP	0,0000351
GO:0022411	cellular component disassembly	BP	0,000038
GO:1901987	regulation of cell cycle phase transition	BP	0,0000386
GO:0099513	polymeric cytoskeletal fiber	CC	0,0000417
GO:0000075	cell cycle checkpoint	BP	0,0000446
GO:0031577	spindle checkpoint	BP	0,0000484
GO:0099512	supramolecular fiber	CC	0,000051
GO:0044772	mitotic cell cycle phase transition	BP	0,0000521
GO:0051321	meiotic cell cycle	BP	0,0000619
GO:0043244	regulation of protein complex disassembly	BP	0,0000619
GO:0005871	kinesin complex	CC	0,0000641
GO:0007088	regulation of mitotic nuclear division	BP	0,0000773
GO:0002520	immune system development	BP	0,0000796
GO:1901990	regulation of mitotic cell cycle phase transition	BP	0,0000813
GO:0051783	regulation of nuclear division	BP	0,0000863
GO:0000788	nuclear nucleosome	CC	0,0000916
GO:0006333	chromatin assembly or disassembly	BP	0,0000937
GO:0051233	spindle midzone	CC	0,000106
GO:0051225	spindle assembly	BP	0,00012
GO:0031497	chromatin assembly	BP	0,000123
GO:0035173	histone kinase activity	MF	0,00015
GO:0016572	histone phosphorylation	BP	0,000179
GO:0030261	chromosome condensation	BP	0,000228
GO:0006334	nucleosome assembly	BP	0,000237

GO:0019900	kinase binding	MF	0,000242
GO:0000790	nuclear chromatin	CC	0,000253
GO:0032467	positive regulation of cytokinesis	BP	0,000289
GO:0034501	protein localization to kinetochore	BP	0,000323
HP:0010976	B lymphocytopenia	hp	0,000402
GO:0045930	negative regulation of mitotic cell cycle	BP	0,000431
GO:0008283	cell proliferation	BP	0,000483
GO:0042127	regulation of cell proliferation	BP	0,000497
GO:0005875	microtubule associated complex	CC	0,000518
GO:0051640	organelle localization	BP	0,000521
GO:0032403	protein complex binding	MF	0,000544
HP:0010975	Abnormality of B cell number	hp	0,000595
GO:0003777	microtubule motor activity	MF	0,000804
GO:0045931	positive regulation of mitotic cell cycle	BP	0,000849
GO:0071168	protein localization to chromatin	BP	0,000882
GO:0008574	ATP-dependent microtubule motor activity, plus-end-directed	MF	0,000882
KEGG:05034	Alcoholism	keg	0,000988
GO:0050000	chromosome localization	BP	0,001
GO:0051303	establishment of chromosome localization	BP	0,001
GO:0007126	meiotic nuclear division	BP	0,0011
GO:0046983	protein dimerization activity	MF	0,00138
GO:0051310	metaphase plate congression	BP	0,00147
GO:0032465	regulation of cytokinesis	BP	0,00153
GO:1903046	meiotic cell cycle process	BP	0,00167
GO:0006342	chromatin silencing	BP	0,00176
GO:0034728	nucleosome organization	BP	0,0019
GO:0019901	protein kinase binding	MF	0,00195
GO:1990939	ATP-dependent microtubule motor activity	MF	0,00202
GO:0051304	chromosome separation	BP	0,00258
GO:0045814	negative regulation of gene expression, epigenetic	BP	0,00258
GO:0046982	protein heterodimerization activity	MF	0,00336
GO:1902850	microtubule cytoskeleton organization involved in mitosis	BP	0,00396
GO:0090307	mitotic spindle assembly	BP	0,00396
GO:0051656	establishment of organelle localization	BP	0,00405
GO:0008092	cytoskeletal protein binding	MF	0,00439
GO:0010948	negative regulation of cell cycle process	BP	0,00443
GO:0097149	centralspindlin complex	CC	0,00486
GO:0044839	cell cycle G2/M phase transition	BP	0,00534
GO:0006259	DNA metabolic process	BP	0,00614
GO:0007080	mitotic metaphase plate congression	BP	0,00631
GO:0040029	regulation of gene expression, epigenetic	BP	0,00669
HP:0002664	Neoplasm	hp	0,00687
HP:0011793	Neoplasm by anatomical site	hp	0,00712
GO:0006468	protein phosphorylation	BP	0,00753
HP:0040088	Abnormal lymphocyte count	hp	0,00799
GO:0051297	centrosome organization	BP	0,00912
HP:0100280	Crohn's disease	hp	0,0102
GO:0007093	mitotic cell cycle checkpoint	BP	0,0128
GO:0051338	regulation of transferase activity	BP	0,0136
GO:0042113	B cell activation	BP	0,015
GO:0033045	regulation of sister chromatid segregation	BP	0,0154
GO:0031023	microtubule organizing center organization	BP	0,0162
GO:1990752	microtubule end	CC	0,0165
GO:1902749	regulation of cell cycle G2/M phase transition	BP	0,0174
GO:0051290	protein heterotetramerization	BP	0,0181
GO:0007127	meiosis I	BP	0,0182
GO:0051988	regulation of attachment of spindle microtubules to kinetochore	BP	0,0186
GO:0007076	mitotic chromosome condensation	BP	0,0186
KEGG:05203	Viral carcinogenesis	keg	0,0186
GO:0000942	condensed nuclear chromosome outer kinetochore	CC	0,0193
GO:0051785	positive regulation of nuclear division	BP	0,0196
GO:0016458	gene silencing	BP	0,0202
HP:0002743	Recurrent enteroviral infections	hp	0,0216

GO:0002521	leukocyte differentiation	BP	0,0223
HP:0004377	Hematological neoplasm	hp	0,0227
GO:0051240	positive regulation of multicellular organismal process	BP	0,023
GO:0000784	nuclear chromosome, telomeric region	CC	0,024
GO:1901988	negative regulation of cell cycle phase transition	BP	0,0257
GO:2000026	regulation of multicellular organismal development	BP	0,0263
GO:0016570	histone modification	BP	0,0265
GO:0031145	anaphase-promoting complex-dependent proteasomal ubiquitin-dependent protein catabolic process	BP	0,0276
GO:0008284	positive regulation of cell proliferation	BP	0,0314
GO:0000086	G2/M transition of mitotic cell cycle	BP	0,0341
GO:0051782	negative regulation of cell division	BP	0,0341
GO:0016569	covalent chromatin modification	BP	0,0354
GO:0016568	chromatin modification	BP	0,0359
GO:1902099	regulation of metaphase/anaphase transition of cell cycle	BP	0,0378
GO:0030071	regulation of mitotic metaphase/anaphase transition	BP	0,0378
GO:0051094	positive regulation of developmental process	BP	0,0391
GO:0034622	cellular macromolecular complex assembly	BP	0,0411
GO:0044784	metaphase/anaphase transition of cell cycle	BP	0,0433
GO:0045840	positive regulation of mitotic nuclear division	BP	0,0433
GO:0007091	metaphase/anaphase transition of mitotic cell cycle	BP	0,0433
HP:0100658	Cellulitis	hp	0,0465
GO:0032133	chromosome passenger complex	CC	0,0477
CORUM:5547	Cdc2-Ccnb1 complex	cor	0,0499
CORUM:5706	CyclinB1-Cdc2 complex	cor	0,0499

Table S5. GO terms of up-regulated genes.

term.id	term.name	domain	p-value
GO:0006955	immune response	BP	4,11E-14
GO:0006952	defense response	BP	1,02E-12
GO:0032101	regulation of response to external stimulus	BP	2,54E-12
GO:0002682	regulation of immune system process	BP	3,24E-12
GO:0002252	immune effector process	BP	2,48E-10
GO:0006954	inflammatory response	BP	9,56E-10
GO:1903034	regulation of response to wounding	BP	1,98E-08
GO:0080134	regulation of response to stress	BP	2,75E-08
GO:0001816	cytokine production	BP	4,49E-08
GO:0050776	regulation of immune response	BP	5,29E-08
KEGG:04060	Cytokine-cytokine receptor interaction	keg	1,22E-07
GO:0002684	positive regulation of immune system process	BP	1,33E-07
GO:0031347	regulation of defense response	BP	1,42E-07
GO:0009611	response to wounding	BP	4,92E-07
GO:0032103	positive regulation of response to external stimulus	BP	5,09E-07
GO:0009986	cell surface	CC	5,93E-07
GO:0001775	cell activation	BP	6,28E-07
GO:0045087	innate immune response	BP	8,4E-07
GO:0009897	external side of plasma membrane	CC	8,52E-07
GO:0051240	positive regulation of multicellular organismal process	BP	0,000002
GO:0001817	regulation of cytokine production	BP	6,47E-06
GO:0009607	response to biotic stimulus	BP	6,48E-06
GO:0051707	response to other organism	BP	9,57E-06
GO:0005615	extracellular space	CC	9,69E-06
GO:0043207	response to external biotic stimulus	BP	9,81E-06
GO:0048584	positive regulation of response to stimulus	BP	1,35E-05
GO:0031349	positive regulation of defense response	BP	1,43E-05
GO:0045321	leukocyte activation	BP	1,43E-05
GO:0004896	cytokine receptor activity	MF	0,000015
GO:0001819	positive regulation of cytokine production	BP	2,11E-05
GO:0098552	side of membrane	CC	2,38E-05
GO:0050900	leukocyte migration	BP	2,83E-05
GO:0060326	cell chemotaxis	BP	3,27E-05
GO:0007155	cell adhesion	BP	6,48E-05
GO:1903036	positive regulation of response to wounding	BP	7,56E-05
GO:0022610	biological adhesion	BP	7,93E-05
GO:0030595	leukocyte chemotaxis	BP	8,73E-05
GO:0050865	regulation of cell activation	BP	9,44E-05
GO:0002366	leukocyte activation involved in immune response	BP	0,000116
GO:0002263	cell activation involved in immune response	BP	0,00013
GO:0002274	myeloid leukocyte activation	BP	0,000157
GO:0050778	positive regulation of immune response	BP	0,000277
GO:0002520	immune system development	BP	0,00029
GO:0002521	leukocyte differentiation	BP	0,000467
GO:0048534	hematopoietic or lymphoid organ development	BP	0,000472
GO:0098542	defense response to other organism	BP	0,000532
GO:0050727	regulation of inflammatory response	BP	0,000568
GO:0030097	hemopoiesis	BP	0,000625
GO:0050786	RAGE receptor binding	MF	0,000863
GO:0002694	regulation of leukocyte activation	BP	0,000992
GO:0046903	secretion	BP	0,00103
GO:0034097	response to cytokine	BP	0,00114
GO:0050777	negative regulation of immune response	BP	0,00124

GO:0002443	leukocyte mediated immunity	BP	0,00128
GO:0002687	positive regulation of leukocyte migration	BP	0,00145
GO:0019221	cytokine-mediated signaling pathway	BP	0,00179
GO:1902533	positive regulation of intracellular signal transduction	BP	0,00184
GO:0050866	negative regulation of cell activation	BP	0,00195
GO:0002683	negative regulation of immune system process	BP	0,00205
GO:0002685	regulation of leukocyte migration	BP	0,00207
GO:0002697	regulation of immune effector process	BP	0,00219
GO:0050921	positive regulation of chemotaxis	BP	0,00225
GO:0097529	myeloid leukocyte migration	BP	0,00233
GO:1902105	regulation of leukocyte differentiation	BP	0,00259
GO:1903706	regulation of hemopoiesis	BP	0,00276
GO:0002764	immune response-regulating signaling pathway	BP	0,00309
GO:0071345	cellular response to cytokine stimulus	BP	0,00362
GO:0009617	response to bacterium	BP	0,00412
GO:0005102	receptor binding	MF	0,00484
GO:1903708	positive regulation of hemopoiesis	BP	0,00489
GO:1902531	regulation of intracellular signal transduction	BP	0,0053
GO:0002444	myeloid leukocyte mediated immunity	BP	0,00545
GO:0009615	response to virus	BP	0,00592
GO:0002237	response to molecule of bacterial origin	BP	0,00612
GO:0002695	negative regulation of leukocyte activation	BP	0,00692
GO:1902107	positive regulation of leukocyte differentiation	BP	0,00692
GO:0010647	positive regulation of cell communication	BP	0,00947
GO:0048520	positive regulation of behavior	BP	0,0118
GO:0002292	T cell differentiation involved in immune response	BP	0,0136
GO:0006935	chemotaxis	BP	0,0142
GO:0042330	taxis	BP	0,0149
GO:0002690	positive regulation of leukocyte chemotaxis	BP	0,0161
GO:1903035	negative regulation of response to wounding	BP	0,0164
GO:0002285	lymphocyte activation involved in immune response	BP	0,0193
GO:0032496	response to lipopolysaccharide	BP	0,0193
GO:0002526	acute inflammatory response	BP	0,0205
GO:0061041	regulation of wound healing	BP	0,0205
GO:0023056	positive regulation of signaling	BP	0,0205
GO:0009967	positive regulation of signal transduction	BP	0,0208
KEGG:04142	Lysosome	keg	0,0241
GO:2000026	regulation of multicellular organismal development	BP	0,0247
GO:0002819	regulation of adaptive immune response	BP	0,0262
GO:0005509	calcium ion binding	MF	0,0288
GO:0050729	positive regulation of inflammatory response	BP	0,0347
GO:0098609	cell-cell adhesion	BP	0,0349
GO:0042060	wound healing	BP	0,0453
GO:0007596	blood coagulation	BP	0,0469
GO:0050878	regulation of body fluid levels	BP	0,047
GO:0002250	adaptive immune response	BP	0,047
GO:0051094	positive regulation of developmental process	BP	0,0483
GO:0032102	negative regulation of response to external stimulus	BP	0,0498
GO:0031232	extrinsic component of external side of plasma membrane	CC	0,0499
CORUM:44	CASK-MINT complex	cor	0,05
